# Network analyses of discrete emotions and social connectedness indicators in anxiety and depressive disorders

**DOI:** 10.1016/j.xjmad.2026.100177

**Published:** 2026-04-01

**Authors:** Ryan C. Shriver, Madeleine Rassaby, Amanda C. Collins, Charles T. Taylor

**Affiliations:** a Department of Psychiatry, University of California San Diego, 9500 Gilman Drive, La Jolla, CA 92093, United States; b Department of Psychiatry, Massachusetts General Hospital, Boston, MA 02114, United States; c Department of Psychiatry, Harvard Medical School, Boston, MA 02114, United States

**Keywords:** Anxiety, Depression, Positive affect, Negative affect, Social connectedness, Network analysis

## Abstract

Low positive affect (PA) and high negative affect (NA) are common features of depressive and anxiety disorders that have been linked to social disconnection. Prior research, however, has examined PA, NA, and social connectedness as global dimensions rather than as systems of interacting discrete emotions and connectedness indicators. This study used network analysis to: (a) examine interrelationships among discrete emotions to identify potentially influential emotions within and across positive and negative emotion communities, and (b) determine which emotions are most strongly linked to indicators of perceived social connection or disconnection. Data were derived from 359 adults with clinically elevated anxiety or depression who completed measures of discrete emotions and social connectedness. We estimated three networks: Model 1 examined associations among positive and negative emotions; Model 2 examined associations among discrete emotions and indicators of social connection; and Model 3 examined associations among discrete emotions and indicators of social disconnection. Model 1 revealed that *hope*, *joy*, and *guilt/shame* were the most central nodes within their respective communities while *sadness* emerged as a key node linking positive and negative emotions. In Model 2, cross-community links were observed between *love* and *feeling understood*, and between *embarrassment* and *comfort around strangers*. In Model 3, the node *guilt/shame* shared a positive association with *poor belonging* while *love* was negatively associated with *lacking brother/sisterhood among friends*. These findings inform how discrete emotions and social experiences relate in anxiety and depressive disorders, pointing to specific emotions and connectedness indicators that may represent promising treatment targets.

## Highlights

• Network analysis mapped links among discrete emotions in anxiety and depression.
• Identified ‘hope’ and ‘sadness’ as potentially influential discrete emotions.
• Examined associations among emotions and social connectedness indicators.
• Found associations between ‘love’ and multiple social connectedness indicators.
• Highlighted emotion and (dis)connectedness targets to improve social well-being.

## 1 Introduction

### 1.1 Affect in anxiety and depression

Low positive affect (PA) and high negative affect (NA) characterize a common affective profile among individuals with depression and anxiety [1], [2], [3]. Extensive factor analytic research has established PA and NA as robust, higher-order orthogonal structures [4], [5] that account for the covariance of individual emotions (e.g. joy, fear, hope). Accordingly, most research has examined PA and NA as global dimensions in relation to depression and anxiety. Emerging data suggests there may also be value in examining how *discrete* positive and negative emotions characterize anxiety and depressive disorders. First, different emotions may serve different functions: for example, love signals an opportunity for affiliation and intimacy - motivating engagement with and responsiveness to close others, whereas pride arises after personal achievement - motivating future goal pursuit and perseverance [6]. Second, discrete emotion profiles may uncover more precise treatment targets and nuanced diagnostic insights. For example, social anxiety symptom severity has been linked to lower levels of pride, beyond other discrete positive emotions and global PA [7] and diminished experiences of specific positive emotions including inspiration, interest, joy, and awe were found to distinguish major depressive disorder (MDD) from social anxiety disorder (SAD; [8]). While this growing body of research underscores the value of investigating discrete emotions in relation to anxiety and depression, little is known about how discrete positive and negative emotions may *interact with one another* within anxiety and depressive disorder samples. Examining these interconnections can reveal how specific emotions may co-occur, reinforce, or inhibit one another, thereby elucidating the mechanisms underlying low PA and high NA in anxiety and depression. Accordingly, **the first aim** of the current study used network analysis to examine the functional connections (i.e., the existence and strength of bidirectional relationships) among discrete positive and negative emotions in individuals with clinically elevated anxiety or depression. In doing so, we sought to identify which individual emotions were most strongly implicated in the affective profile underlying anxiety and depression, and to discern potential targets for intervention.

### 1.2 Social connectedness in anxiety and depression

In anxiety and depressive disorders, low PA and high NA often co-occur with social disconnection, suggesting an interplay between emotions and interpersonal functioning [9], [10]. Individuals diagnosed with these conditions commonly report greater loneliness, reduced social engagement, and lower perceived support [11], [12], [13], [14]. Intervention studies further indicate a bidirectional relationship between affect and social connectedness. One study in individuals with anxiety or depressive disorders found that performing acts of kindness (e.g., helping others) significantly reduced NA [15] and two others found that increasing PA led to subsequent increases in social connectedness [10], [16]. Despite these findings, most studies conceptualize PA, NA, and social connectedness as global latent constructs rather than discrete emotions and specific indicators of connectedness. Because *social connectedness* represents a multidimensional construct (e.g., perceived support, relational connectedness, isolation; [17], [18]), the present study adopted a more fine-grained, item-level approach. By leveraging network analysis, **the second aim** of the current study examined associations between individual emotions (e.g., embarrassment, love, joy) and indicators of perceived social connection (e.g., feeling understood, feeling close to others) and disconnection (e.g., viewing oneself as a loner) to identify which are most strongly linked and may therefore serve as intervention targets.

### 1.3 Network analysis

The current study used network analysis to clarify the functional connections among discrete features of PA, NA, and social connectedness. Network analysis offers a complementary framework for understanding psychopathology by focusing on the dynamic interactions among individual components (e.g. discrete emotions) rather than focusing solely on the aggregate latent constructs they comprise [19], [20], [21]. Implementing this perspective in the present context, discrete emotions and social connectedness indicators may function as interdependent elements that collectively generate and sustain broader patterns of low PA, high NA, and interpersonal difficulties [21]. Importantly, while the cross-sectional design of the current study precludes the identification of causal direction, network analyses provide a valuable map of functional connectivity that can generate hypotheses for future studies to test temporal or causal relationships [22]. Such insights may add nuance to our current understanding of broader affective dimensions and ultimately guide more precise interventions that prioritize specific emotions with the potential to increase PA, reduce NA, and enhance social connectedness.

To evaluate the potentially self-reinforcing relationships among discrete positive emotions, negative emotions, and social connectedness indicators, we used regularized partial correlation networks [23]. In these networks, two nodes (i.e., discrete emotions) may be visually connected via an edge; that edge represents a partial correlation among those nodes when controlling for all other variables in the network. Importantly, edges are “undirected” such that we were unable to determine whether node A causes node B, node B causes node A, or if the direction of influence goes both ways [24]. This approach yielded statistical outcomes (centrality measures) that identified which discrete emotions had the greatest potential influence on others in their community (e.g., heightened joy associated with the activation of other PA emotions) or across communities (e.g., heightened joy associated with the deactivation of NA emotions, heightened shame associated with decreased comfort around others). Recent studies implementing this approach to model discrete emotions in psychopathology have generated several insights. In a network analysis of eating disorder symptoms and discrete emotions, the nodes *distress* and *fear* were most central among the affect community, suggesting that they may uniquely influence other emotional states [25]. Another study found that for individuals diagnosed with schizophrenia, *shame* was highly central among other NA nodes, and a greater degree of centrality for *shame* was associated with increases in positive symptoms like auditory hallucinations and delusions. Based on these findings, the authors suggested adapting interventions to target specific discrete emotions depending on the prominence of positive or negative symptoms [26].

### 1.4 Current study

To our knowledge, the network analysis approach has not yet been applied to discrete emotions and social connectedness indicators in adults with clinically elevated anxiety and depression. Therefore, the present study represents an initial step toward identifying the most influential discrete emotions and social connectedness indicators in this population. Targeting these components in treatment may generate a cascade of improvements more efficiently than focusing on less central elements [19], [21]. For example, if gratitude emerged as highly influential, interventions could deliberately incorporate gratitude practice to enhance therapeutic outcomes**.**

The current study aimed to: (a) identify connections among discrete emotions both within communities (e.g., PA–PA and NA–NA) and across communities (e.g., PA–NA) to determine which emotions have the potential to influence one another, and (b) identify discrete emotions linked to individual indicators of social connectedness to determine which specific emotions have the strongest potential impact on social connectedness and vice versa. To address these aims, we conducted a secondary analysis of previously collected self-report data - including PA, NA, and social connectedness indicators - drawn from five clinical trials in individuals with clinically elevated anxiety or depression. Three network models were estimated: Model 1 included only PA and NA items to explore intra- and cross-valence connectivity. Models 2 and 3 built upon the first by introducing individual connectedness indicators to examine the associations between discrete emotions and perceived social connection (Model 2) and disconnection (Model 3). These analyses were exploratory, as we did not have specific hypotheses regarding how these psychosocial components would interact.

## 2 Method

### 2.1 Participant enrollment

Participants were enrolled in the context of five parent clinical trials (National Library of Medicine [NLM], ClinicalTrials.gov Identifiers: NCT02136212, NCT02330744, NCT02330627, NCT03196544, & NCT04945239) that, respectively, selected for participants with (1) a current principal diagnosis of social anxiety disorder (SAD) defined using the Mini International Neuropsychiatric Interview (MINI Version 5.0.0; [27]) and Liebowitz Social Anxiety Scale (LSAS; [28]) score ≥ 50 (n = 91) between April 2013 and July 2016; (2) a current principal diagnosis of major depressive disorder (MDD) defined using the MINI Version 6.0.0 [27] and 9-item Patient Health Questionnaire (PHQ-9; [29]) score ≥ 10 (n = 42) between July 2014 and April 2017; (3) clinically elevated depression defined by PHQ-9 score ≥ 10 and/or clinically elevated anxiety defined by Overall Anxiety Severity and Impairment Scale (OASIS; [30]) ≥ 8 (n = 37) between April 2014 and July 2017; (4) clinically elevated depression defined by PHQ-9 score ≥ 10 and/or clinically elevated anxiety defined by OASIS score ≥ 8, disrupted social functioning defined by the Sheehan Disability Scale-Social Domain (SDS-Social; [31]) score ≥ 5, and social disconnection defined by Social Connectedness Scale Revised (SCS-R; 32]) score < 90 (n = 70) between April 2018 and October 2019; (5) clinically elevated depression defined by PHQ-9 score ≥ 10 and/or clinically elevated anxiety defined by OASIS score ≥ 8, disrupted social functioning defined by the Sheehan Disability Scale-Social Domain (SDS-Social; [31]) score ≥ 5, and social disconnection defined by Social Connectedness Scale Revised (SCS-R; [32]) score < 90 (n = 119) between March 2021 and September 2023. Although trials 4 and 5 explicitly required disrupted social functioning and perceived social disconnection for inclusion, participants across all trials exhibited comparable scores on the SDS-S and SCS-R (see Supplementary materials).

Participants between the ages of 18 and 55 years were recruited through clinical referrals as well as posted announcements in community and online settings (e.g., Instagram, Reddit, BuildClinical, ResearchMatch.org). Exclusion criteria were defined by the parent study and used to ensure that participants could safely complete the procedures and to minimize confounding interpretation of our findings: (a) current or recent use of psychotropic or pharmacological treatments that could affect brain functioning (e.g., SSRIs, benzodiazepines, anxiolytics, antidepressants); (b) concurrent psychotherapy or empirically supported treatments for anxiety or depression (e.g., cognitive behavioral therapy; participants meeting 12-week stability were permitted); (c) current active suicidal ideation, particularly with intent or plan; (d) history of major neurological disorder or moderate to severe traumatic brain injury; (e) severe or unstable medical conditions that might be negatively impacted by participation in study procedures; (f) moderate to severe alcohol or marijuana use disorder (past year); or mild to severe substance use disorder (all other drugs; past year); (g) bipolar 1 or psychotic disorders; (h) non-correctable vision or hearing problems (some assessments required intact sensory functioning); (i) inability to complete the assessment batteries or treatment sessions; (j) inability to speak or understand English; (k) characteristics that would compromise MRI safety (e.g., metal devices or implant in the body). Following eligibility assessment, participants who met inclusion criteria were scheduled to complete the baseline behavioral assessment (including Modified Differential Emotions Scale and Social Connectedness Scale Revised, discussed below). Diagnostic interviews for sample inclusion were conducted using the MINI (version dependent on parent trial; [26]) by a PhD-level clinician, PhD student in clinical psychology, or post-baccalaureate clinical research coordinator, all of whom received extensive training on the interview protocols.

### 2.2 Participants

All participants who completed the baseline assessment of their respective parent trial were included in the current study for a total of 359 participants. Demographic characteristics were as follows: age (*M* = 27.74, SD = 9.12), gender (227 female, 125 male, 7 other), race (43% White, 19% Asian, 18.1% Black, 3.4% Pacific Islander, 9.5% other, 7% declined to respond), and ethnicity (24% Hispanic). The majority of participants presented with clinically elevated symptoms of depression and/or anxiety. See Table 1.

**Table 1:** Diagnostic measure descriptives.

**Principal Diagnosis** MDD GAD SAD	**n (%)** 234 (65.2%) 274 (76.3%) 239 (66.6%)	
**Variable** OASIS (n = 322) PHQ-9 (n = 323)	**M (SD)** 10.44 (3.2) 12.69 (5.3)	**Range** 0–19 0–27

*Note.* MDD = Major depressive disorder; GAD = Generalized anxiety disorder; SAD = Social anxiety disorder; OASIS = Overall Anxiety Severity and Impairment Scale; PHQ-9 = 9-item Patient Health Questionnaire. The OASIS and PHQ-9 were not administered until partway through data collection for study 1 (NCT02136212).

### 2.3 Measures

**Positive and Negative Emotions**. Participants completed the 20-item Modified Differential Emotions Scale (mDES; [33]) to assess a broad array of discrete positive (e.g., joy, love, serenity) and negative emotions (e.g., sad, embarrassed, stressed). Participants were asked to indicate how often they experienced each emotion *during the past week*. Responses to each item were rated on a 5-point Likert scale: (0) Never, (1) Rarely, (2) Some of the time, (3) Often, (4) Most of the time. In the current sample, the mDES demonstrated strong internal consistency, with Cronbach’s *α* = .906 for the PA subscale and *α* = .849 for the NA subscale. See Table S1 for emotion descriptors.

**Social Connectedness.** Social connectedness was assessed using the 20-item Social Connectedness Scale-Revised (SCS-R; [32]). The SCS-R measures social connectedness as a psychological sense of belonging, or as one’s cognition of interpersonal closeness with the surrounding social world. The scale consists of 20 items (10 positively-worded and 10 negatively-worded) rated on a 6-point Likert scale (1 = *strongly disagree* to 6 = *strongly agree*). Participants were asked to “rate the degree to which you agree or disagree with each statement”; no time frame was provided to anchor responses. Sample items include “I see people as friendly and approachable”; “I catch myself losing a sense of connection with society”; and “I feel comfortable in the presence of strangers.” The SCS-R is a widely accepted measurement tool for evaluating social connectedness and displays high internal consistency, test-retest reliability, and convergent validity [32]. In the current sample, internal consistency for the total scale was strong (*α* = .886).

**Anxiety and Depression Symptoms.** Anxiety symptoms were assessed using the Overall Anxiety Severity and Impairment Scale (OASIS; [30]), a 5-item scale that measures the frequency and severity of anxiety symptoms *during the past 2 weeks*. Internal consistency for the OASIS scale in the current sample was strong (*α* =.815). Depressed mood was assessed using the Patient Health Questionnaire (PHQ-9; [29]), a 9-item scale that measures the presence of symptoms *during the past 2 weeks* (see Footnote 1). In the current sample, internal consistency for the PHQ-9 was strong (*α* =.813).

### 2.4 Procedure

This study was conducted within the context of larger treatment trials for anxiety and depressive disorders. Study procedures for all trials were approved by the Human Research Protections Program at the University of California San Diego. After receiving detailed information about the research, participants provided written informed consent and then completed diagnostic and symptom eligibility assessments. Those who met eligibility criteria subsequently completed a battery of self-report measures, as described above, prior to beginning treatment.

### 2.5 Data analytic plan

#### 2.5.1 Missing data

Descriptive frequency and internal consistency analyses were conducted using SPSS (version 29.0), whereas all network analyses were performed in RStudio (version 2025.09.1). Among the variables included in our network models, 0.13% of data were missing. To maintain the full analytic sample and prevent loss of statistical power, multiple imputation (m = 20) was performed using the *mice* package in R with Predictive Mean Matching (PMM). Given the extremely low rate of missingness, the first completed dataset was extracted for subsequent network analyses [34].

#### 2.5.2 Node selection

To fulfill our primary study objectives, we estimated and evaluated three separate network models. **Our first model** consisted of 10 PA and 10 NA items from the mDES, representing an array of discrete emotional states. As commonly done with network analysis, items were examined to minimize redundancy using the *networktools* package [35]. The Hittner method [36] was used to compare correlations between each item. The threshold was set to 0.25 and corMin to 0.55, such that any items with less than 25% of significantly different correlations and a zero-order correlation of 0.55 or above would be considered redundant. Any two items that were identified as redundant were combined using the *reduce_net* function from the *networktools* package [35]. **Our second model** built upon the first by adding the 10 positively-worded items from the SCS-R to those items from the first model. This model allowed us to interpret the associations among discrete emotions and individual indicators of perceived social connection. **Our third model** built upon the first by adding the 10 negatively-worded items from the SCS-R to those items from the first model. This model allowed us to interpret the associations among discrete emotions and individual indicators of perceived social disconnection. All SCS-R items in Model 2 and 3 were evaluated for redundancy using the same methods and criteria as in Model 1. The decision to model the positively and negatively-worded SCS-R items separately was guided by recommendations to limit the number of nodes based on sample size (N = 359) to maximize network stability, accuracy, and interpretability [37].

#### 2.5.3 Network estimation

All network models were created using the *estimateNetwork* function in the *bootnet* package [35]. Each network was estimated via a Graphical Gaussian Model whereby each item represents a “node” and each partial correlation between two nodes represents an “edge.” Each edge, viewed as a line when plotted, appears thicker for a strong correlation and thinner for a weak correlation. Additionally, a blue line represents a positive relationship among two nodes while a red line indicates a negative relationship. To reduce spurious, false-positive edges, we utilized the Extended Bayesian Information Criterion (EBIC; [38]) and the least absolute shrinkage and selection operator (LASSO; [39]). The EBIC is a method for selecting the hyperparameter gamma (γ) used in LASSO, which controls the degree to which a network will either be sparse (higher γ) or dense (lower γ). Graphical LASSO then shrinks small estimates to zero and thus limits the number of edges present, resulting in a more interpretable network [40].

#### 2.5.4 Centrality

To quantify the importance of the nodes in our networks and evaluate the relationships among them, we estimated a number of network statistics. Each pair of nodes (e.g., joy – serenity) that maintain a significant edge following regularization were assigned a value, or *edge weight*, with those of higher magnitude reflecting stronger edge weights*.* Expected influence (EI) simply sums the values of all edges connected to an individual node, thereby providing a measure of overall positive connectivity in networks with both positive and negative edges [41]. An individual node’s influence on the nodes it is directly connected to and subsequently indirectly connected to is represented via one-step and two-step EI, respectively. We opted to evaluate node EI, rather than node strength, as our main centrality measure given that our models contain both positive and negative edges, and node strength only sums the absolute values of edge weights [41]. Importantly, the absolute magnitude of raw EI values is inherently influenced by the size (number of nodes included) and density of the network. Specifically, as the number of nodes and potential edges increases, the theoretical upper and lower bounds for the sum of edge weights also increases.

#### 2.5.5 Bridge analysis

To better understand the cross-community relationships between the different constructs in our models (i.e., PA nodes sharing edges with NA nodes), we examined the extent to which, if any, of the nodes bridged to those of other constructs. To do so, we utilized the *bridge* function in the *networktools* package, which sums the edge-weights of a given node to nodes in different communities [35]. Of note, the communities in our models were defined a priori based on construct membership (positive affect, negative affect, perceived social connection, and perceived social disconnection). These analyses were pertinent to the current study, as nodes with higher bridge expected influence (bEI) to other communities may be highly influential within that community [42]. As with EI, both one- and two-step bEI can be calculated.

#### 2.5.6 Edge-weight accuracy, centrality stability, and centrality significance

Following network estimation, we used bootstrapping methods to determine the accuracy, stability, and significance of the network parameters and centrality statistics [23]. Accuracy was examined by evaluating the 95% CI for each edge-weight, constructed via non-parametric bootstrapping [43]. Here, wide CI’s indicate that many of the edge weights do not significantly differ from one another and it may be difficult to interpret the true strength of an edge [40]. Additionally, we used the centrality bootstrapped difference to assess whether nodes with the highest EI or bEI were significantly different from other nodes [37]. Demonstrating that a node is reliably stronger than others helped strengthen our confidence that it may play a uniquely influential role in the network. The case-dropping bootstrap evaluates the correlation between original centrality indices (i.e., expected influence) and the centrality indices based on a subset of the original data. If the correlation stability coefficient (CS-coefficient) drops dramatically as the subset sample gets smaller, the centrality estimate is likely unstable. This method of bootstrapping has led to a standardized view that the CS-coefficient should not be below 0.25 and should ideally be above 0.5 [23], [40]. CS-coefficients ranging from 0.25 to 0.5 are therefore considered ‘acceptable’ but > 0.5 is preferred.

## 3 Results

### 3.1 Node selection

All 20 mDES items were examined for redundancy before running subsequent analyses using the methods stated in Section 2.5.2. In Model 1, the NA nodes *guilt* and *ashamed* were flagged for redundancy and combined into one node (*guilt/shame*) as were the NA nodes *hatred* and *disgust* (*hatred/disgust*). The PA nodes *interested* and *amused* were flagged for redundancy and combined into one node (*interest/amused*), thus 17 total nodes were included in Model 1. In Model 2, none of the SCS-R items were flagged for redundancy and therefore 27 total nodes were included in the model. See Table 2 for a description of the SCS-R nodes included in Model 2. In Model 3, the SCS-R nodes ‘*I feel like an outsider*’ and ‘*I feel distant from people*’ were flagged for redundancy and combined into one node (*SC19*), thus 26 total nodes were included in Model 3. See Table 3 for a description of the SCS-R nodes included in Model 3.

**Table 2:** Social connection (SCS-R) items included in model 2.

SC1	I feel comfortable in the presence of strangers.
SC2	I am in tune with the world.
SC3	I fit in well in new situations.
SC4	I feel close to people.
SC5	I see people as friendly and approachable.
SC6	I feel understood by the people I know.
SC7	I am able to relate to my peers.
SC8	I find myself actively involved in people’s lives.
SC9	I am able to connect with other people.
SC10	My friends feel like family.

**Table 3:** Social disconnection (SCS-R) items included in model 3.

SC11	Even among my friends, there is no sense of brother/sisterhood.
SC12	I feel disconnected from the world around me.
SC13	Even around people I know, I don’t feel that I really belong.
SC14	I have little sense of togetherness with my peers.
SC15	I catch myself losing a sense of connectedness with society.
SC16	I see myself as a loner.
SC17	I don’t feel related to most people.
SC18	I don’t feel I participate with anyone or any group.
SC19	I feel like an outsider; I feel distant from people.

### 3.2 Model 1: discrete emotions in anxiety & depression

The network structure of PA and NA items among our sample is shown in Fig. 1a. Network stability was above recommended thresholds for Model 1 (CS-expected influence = 0.749, CS-bridge expected influence = 0.67, CS-edge weight = 0.749). Bootstrapping results are included in the Supplementary materials. Raw one-step EI values for all Model 1 nodes ranged from 0.28 to 1.15. The nodes *hope* (EI_1_ = 1.15; EI_2_ = 2.05), *inspired* (EI_1_ = 1.02; EI_2_ = 1.93), *guilt/shame* (EI_1_ = 1.00; EI_2_ = 1.61), and *joy* (EI_1_=0.99; EI_2_ = 1.74) emerged as having the strongest one and two-step EI within their respective communities. Fig. 1b displays the one-step and two-step EI of each node. Bootstrapped difference tests revealed that EI values for the nodes *hope* and *inspired* were significantly higher than 75% of all other nodes (Fig. S2).

**Fig. 1a:**
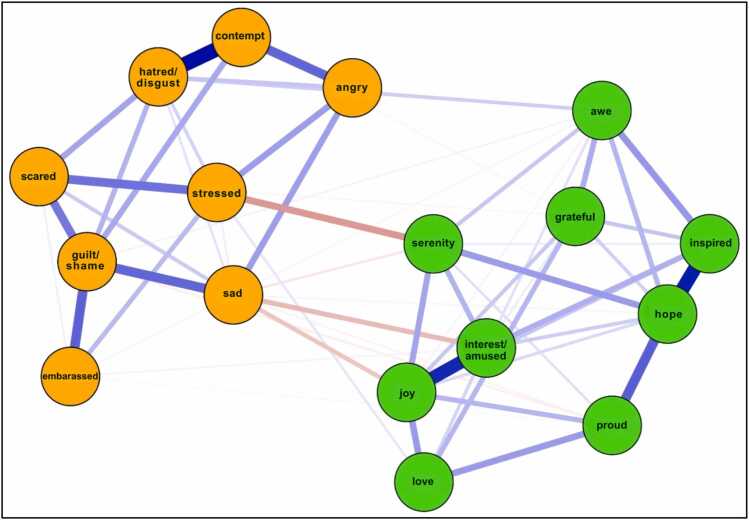
Model 1: network of discrete emotions. Green nodes represent positive emotions. Orange nodes represent negative emotions.

**Fig. 1b:**
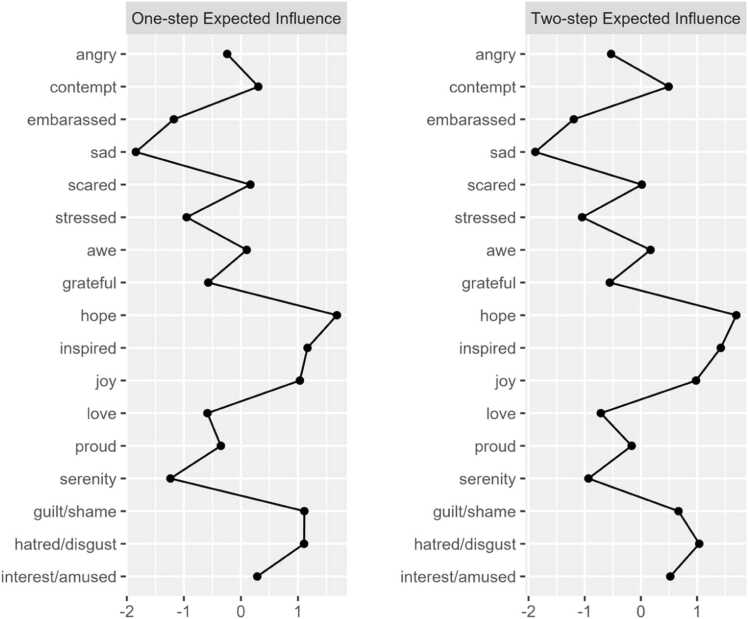
1 and 2-Step EI values (as z-scores) for all nodes in Model 1.

Regarding cross-community influence, the nodes *sad* (bEI_1_ = −0.33; bEI_2_ = −0.63), *serenity* (bEI_1_ = −0.23; bEI_2_ = −0.39), and *awe* (bEI_1_ = 0.15; bEI_2_= 0.24) emerged as having the strongest one and two-step bEI. Fig. 1c displays the one-step and two-step bEI of each node. Cross-community edge weights for the nodes *stressed*–*serenity* (EW = −.18) and *sad*–*interested/amused* (EW = −.12) were the strongest among all other edges connecting NA and PA. Bootstrapped difference tests revealed that bEI for the node *sad* was significantly higher than all other nodes except for *serenity* in Model 1 (Fig. S3).

**Fig. 1c:**
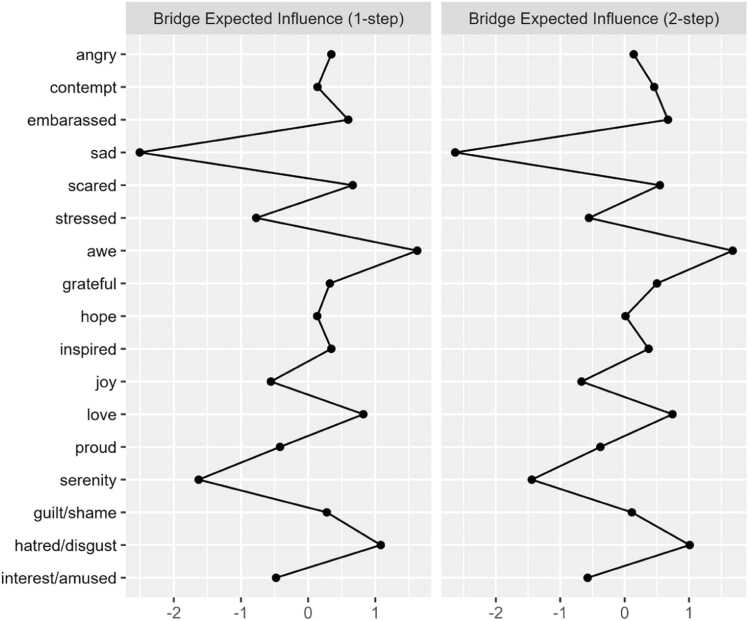
1 and 2-Step bEI values (as z-scores) for all nodes in Model 1.

### 3.3 Model 2: discrete emotions and perceived social connection in anxiety and depression

The network structure of PA, NA, and the positively-worded SCS-R items among our sample in Model 2 is shown in Fig. 2a. Network stability was above the recommended threshold for Model 2 (CS-expected influence = 0.52, CS-bridge expected influence = 0.52, CS-edge weight = 0.52). Bootstrapping results are included in the Supplementary materials. Raw one-step EI values for all Model 2 nodes ranged from 0.21 to 1.23. As seen in Model 1, the nodes *hope* (EI_1_ = 1.23; EI_2_ = 2.23), *inspired* (EI_1_= 1.09; EI_2_ = 2.07), *joy* (EI_1_ = 1.04; EI_2_ = 1.88), and *guilt/shame* (EI_1_ = 0.95; EI_2_ = 1.43) emerged as having the strongest one and two-step EI. Fig. 2b displays the one-step and two-step EI of each node.

**Fig. 2a:**
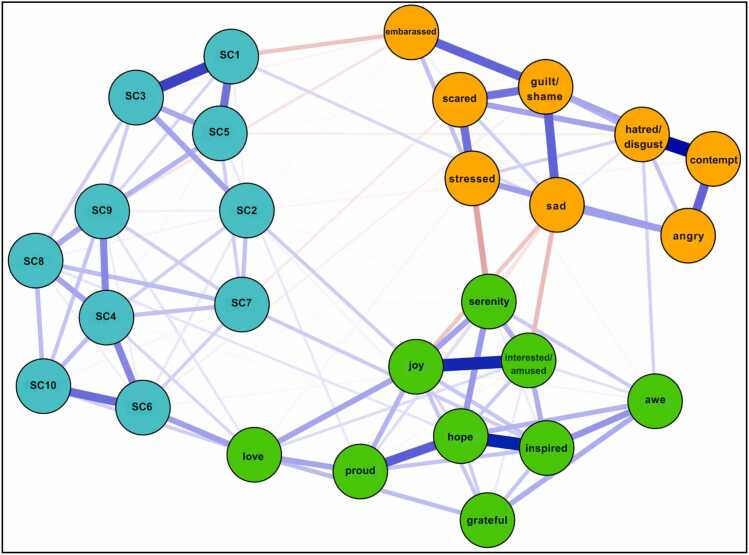
Model 2: network of positively worded SCS-R items and discrete emotions. Blue nodes represent indicators of perceived social connection. Green nodes represent positive emotions. Orange nodes represent negative emotions. See the key in Table 2 for SCS-R node names.

**Fig. 2b:**
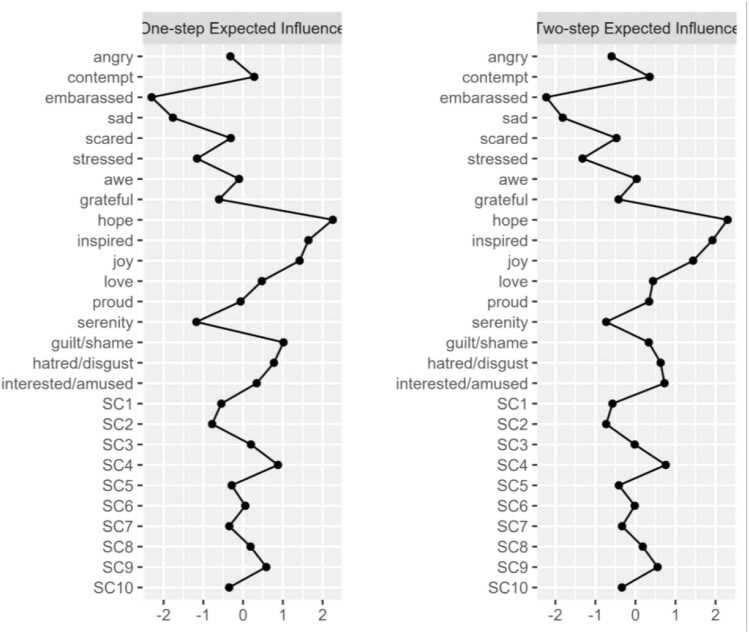
1 and 2-Step EI values (as z-scores) for all nodes in Model 2.

Regarding cross-community influence, the nodes *love* (bEI_1_ = 0.37; bEI_2_ = 0.61), *sad* (bEI_1_ = −0.25; bEI_2_ = −0.53), and *serenity* (bEI_1_ = −0.17; bEI_2_ = −0.27) emerged as having the strongest one and two-step bEI. Among the social connection community, the node *SC6* (‘I feel understood by the people I know’; bEI_1_ = 0.12; bEI_2_ = 0.21) emerged as having the strongest one and two-step bEI. Fig. 2c displays the one-step and two-step bEI of each node. Cross-community edge weights for the nodes *stressed*–*serenity* (EW = −.15) and *sad*–*interest/amused* (EW = −.11) were the strongest among all other edges connecting NA and PA. Cross-community edge weights for the nodes *love–SC6* (‘I feel understood by the people I know’; EW = 0.15) and embarrassed–*SC1* (‘I feel comfortable in the presence of strangers’; EW = −0.10) were the strongest among all other edges connecting NA or PA to SCS-R. *Love* was also positively associated with the social connection community via nodes *SC10* (‘My friends feel like family’; EW = .08), *SC4* (‘I feel close to people’; EW = .07), and *SC9* (‘I am able to connect with other people’; EW = .05). Bootstrapped difference tests revealed that bEI for the node *love* was significantly different from all other nodes in Model 2 (Fig. S9).

**Fig. 2c:**
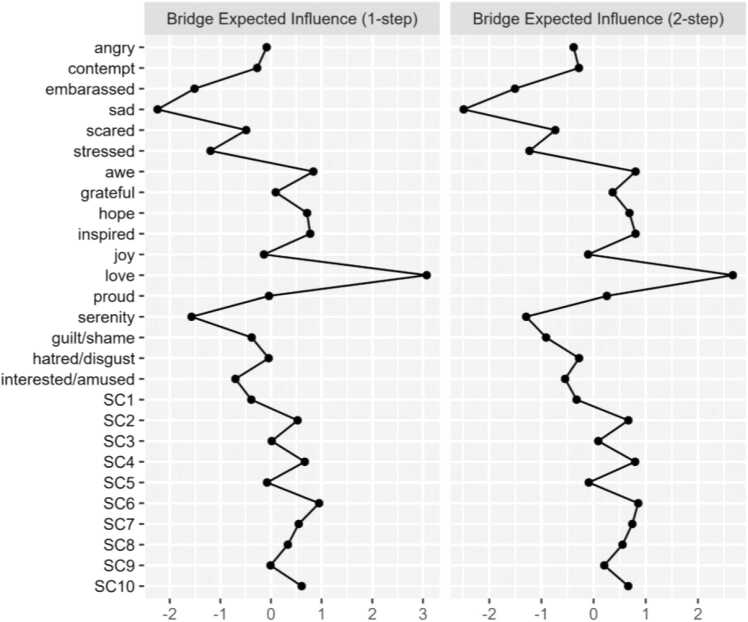
1 and 2-Step bEI values (as z-scores) for all nodes in Model 2.

### 3.4 Model 3: discrete emotions and perceived social disconnection in anxiety and depression

The network structure of PA, NA, and the negatively-worded SCS-R items among our sample in Model 3 is shown in Fig. 3a. Network stability was above the recommended threshold for Model 3 (CS-expected influence = 0.59, CS-bridge expected influence = 0.52, CS-edge weight = 0.59). Bootstrapping results are included in the supplementary materials. Raw one-step EI values for all Model 3 nodes ranged from 0.22 to 1.09. The nodes *guilt/shame* (EI_1_ = 1.09; EI_2_ = 1.77), *hope* (EI_1_ = 1.07; EI_2_ = 1.87), *SC19* (Combined node: ‘I feel distant from people’ & ‘I feel like an outsider’; EI_1_ = 1.05; EI_2_ = 1.80), and *hatred/disgust* (EI_1_ = 1.04; EI_2_ = 1.82) emerged as having the strongest one and two-step EI. Fig. 3b displays the one-step and two-step EI of each node.

**Fig. 3a:**
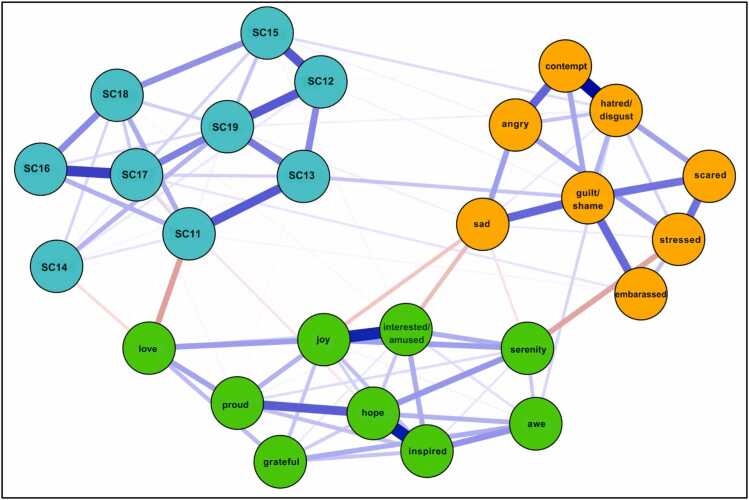
Model 3: network of negatively worded SCS-R items and discrete emotions. Blue nodes represent indicators of perceived social disconnection. Green nodes represent positive emotions. Orange nodes represent negative emotions. See the key in Table 3 for SCS-R node names.

**Fig. 3b:**
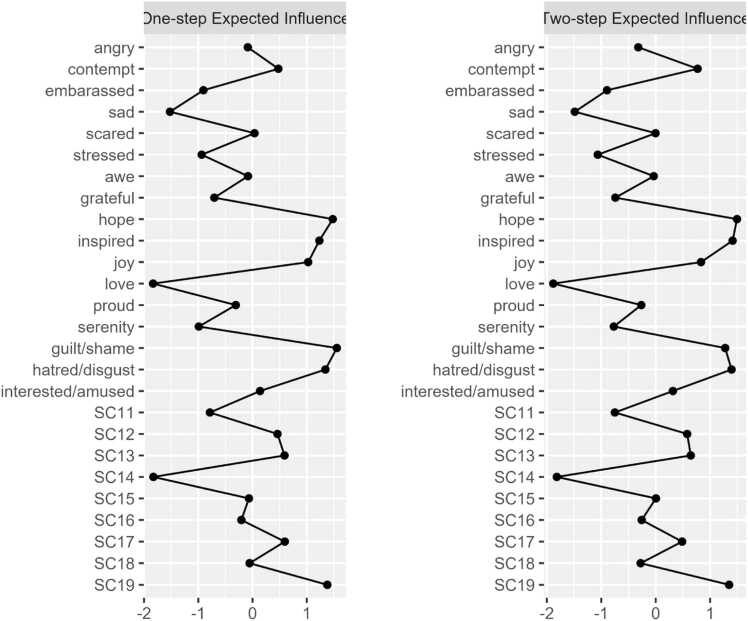
1 and 2-Step EI values (as z-scores) for all nodes in Model 3.

Regarding cross-community influence, the nodes *sad* (bEI_1_ = −0.27; bEI_2_ = −0.45), *love* (bEI_1_ = −0.25; bEI_2_ = −0.46), and *serenity* (bEI_1_ = −0.21; bEI_2_ = −0.37) emerged as having the strongest one and two-step bEI. Among the social disconnection community, the nodes *SC11* (‘Even among my friends, there is no sense of brother/sisterhood’; bEI_1_ = −0.13; bEI_2_ = −0.19) and *SC14* (‘I have little sense of togetherness with my peers’; bEI_1_= −0.12; bEI_2_ = −0.18) emerged as having the strongest one and two-step bEI. Fig. 3c displays the one-step and two-step bEI of each node. As seen in Model 2, cross-community edge weights for the nodes *stressed*–*serenity* (EW = −.15) and *sad*–*interest/amused* (EW = −.11) were the strongest among all other edges connecting NA and PA. Cross-community edge weights for the nodes *love–SC11* (‘Even among my friends, there is no sense of brother/sisterhood’; EW = −0.16) and *guilt/shame*–*SC13* (‘Even around people I know, I don’t feel that I really belong’; EW = 0.09) were the strongest among all other edges connecting NA or PA to SCS-R.

**Fig. 3c:**
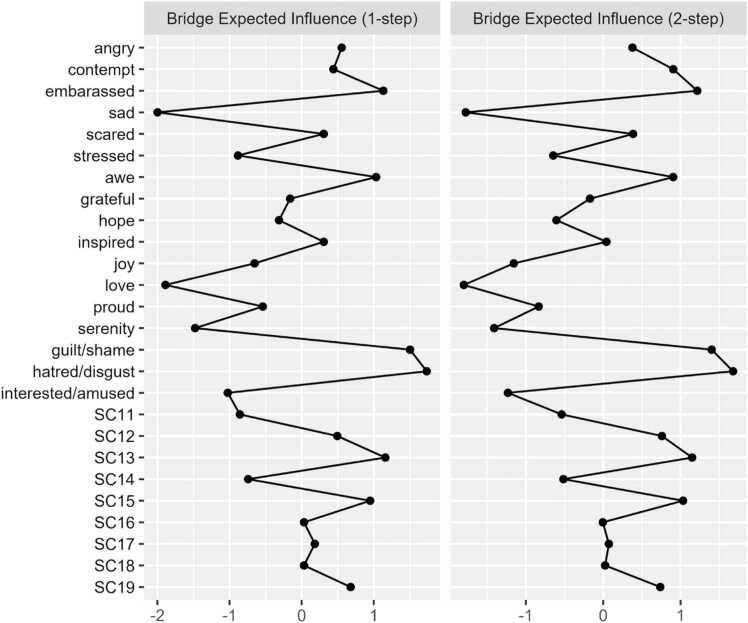
1 and 2-Step bEI values (as z-scores) for all nodes in Model 3.

## 4 Discussion

The current study employed network analysis to examine how discrete emotions and individual indicators of perceived social connection and disconnection relate to one another in the context of clinically elevated anxiety and depression. Our first model, consisting of only discrete emotions, revealed that *hope*, *inspired*, *joy,* and *guilt/shame* were the most central discrete emotions within their respective communities. *Sad* and *serenity* emerged as key bridge nodes linking positive and negative emotions, with *sad* showing a potential inhibitory influence on PA and *serenity* showing a potential inhibitory influence on NA. In our second model with discrete emotions and indicators of perceived social connection, *love*, *sad*, and *serenity* were the most influential bridge nodes from the affect communities while *SC6* (‘I feel understood by the people I know’) was the most influential bridge node from the social connection community. *Love* emerged as a key bridge node linking PA to the social connection community, showing a strong potential positive influence on perceived social connection. In our third model with discrete emotions and indicators of perceived social disconnection, *love* emerged as a key bridge node linking PA to the social disconnection community via a negative association with *SC11* (‘Even among my friends, there is no sense of brother/sisterhood’). Additionally, the NA node *guilt/shame* shared a notable positive association with *SC13* (‘Even around people I know, I don’t feel that I really belong’).

### 4.1 Model 1: discrete emotions in anxiety & depression

The most central nodes in Model 1 represent the discrete emotions that are most strongly interconnected among individuals with clinically elevated anxiety and depression. The nodes *hope*, *joy*, *inspired,* and *guilt/shame* exhibited the strongest expected influence within their respective communities, suggesting that fluctuations in these emotions may have the greatest potential to influence other emotions. Of note, within the PA community, *hope*, *joy*, and *inspired* shared only positive edge weights with other PA emotions (potentially upregulating PA). Similarly, within the NA community, the node *guilt/shame* shared only positive edge weights with other NA emotions (potentially upregulating NA). In observing the nodes surrounding *hope* in the PA community, we noted that experiencing hope was positively associated with experiencing pride (*proud*), inspiration (*inspired*), and peaceful contentment (*serenity*). Thus, an intervention approach to increase experiences of hope may concurrently increase experiences of broader PA, including pride, inspiration, and serenity. Based on the nodes surrounding *guilt/shame* in the NA community, we saw that experiences of guilt and shame were positively associated with experiencing embarrassment (*embarrassed*), fear (*scared*), and sadness (*sad*). Therefore, an intervention approach to mitigate experiences of guilt and shame may concurrently decrease experiences of broader NA, including embarrassment, fear, and sadness. Importantly, the nodes *hope* and *guilt/shame* showed primarily near-zero cross-community edge weights (*hope* was not connected to the NA community; *guilt/shame* was weakly connected to the PA community via *awe*). So, despite being influential within their own communities, it is possible that any intervention targeting hope, guilt, and/or shame may not directly influence the opposing affective community (e.g., targeting hope may not directly target shame).

#### 4.1.1 Hope emerges as a positive mechanism of change

Our results add to the existing literature pointing to hope as a promising mechanism of change in the treatment of anxiety and depressive disorders [44], [45]. Taken with results from previous studies, the present findings indicate that hope may be a pertinent intervention target for treatments aimed at increasing PA and alleviating symptoms in such populations [46]. One study examining hope in the context of clinically elevated anxiety found that undergoing cognitive behavior therapy (CBT) resulted in moderate to large intraindividual increases in hope and that changes in hope predicted improvements in both self-reported and clinician-rated anxiety [45]. Regarding the maintenance of NA, our results align with past research highlighting the unique role of shame, particularly in those with social anxiety disorder (SAD; [47]). Our findings point to shame as a potentially important intervention target, as the maintenance of such negative self-perceptions may lead one to view themselves as fundamentally flawed, inferior, and inadequate [48], [49]. Consistent with this interpretation, compassion-focused and cognitive-behavioral interventions can explicitly target shame and guilt by addressing maladaptive self-criticism and negative self-beliefs while fostering self-compassion and cognitive reappraisal [50], [51], [52].

#### 4.1.2 Cross-valence connectivity

Upon investigating cross-community connectivity, we identified the nodes *sad*, *serenity*, and *awe* as central bridge items. Interestingly, *awe* shared positive associations with the NA community, indicating *awe* could be considered a response to shocking or surprising negative events. The node *serenity* was negatively associated with the node *stressed*, a finding that aligns with previous work positioning calmness and relaxation as emotional states that can “undo” the physiological and psychological effects of stress, thereby serving as natural buffers against arousal [53]. Importantly, the node *sad* stood out as having a significantly stronger bridge-EI relative to all but one other node (*serenity*). As previously noted, nodes with higher bridge EI to opposing communities may be highly influential in that community [42]. Because the node *sad* shares only negative edge weights with the PA community, we can infer that its activation may result in the deactivation of numerous discrete positive emotions, particularly those with which it shares an edge (e.g., joy, interest, amusement, serenity). The finding builds upon existing literature highlighting the role of sadness in clinical anxiety and depression.

One network analysis modeling both DSM and non-DSM symptoms of depression identified ‘sad mood’, ‘loss of interest’, and ‘loss of pleasure’ as highly central and strongly interconnected nodes [54]. Another network analysis investigating relationships among depression and anxiety symptoms found that ‘sad mood’ was the most central symptom across all centrality indices [55]. The extant literature has also pointed to sad mood, relative to other depressive symptoms, as the most debilitating for psychosocial functioning [56]. Furthermore, the notable cross-valence connectivity of the node *stressed* in our model aligns with findings that ‘worry’ and ‘inability to relax’ are among the most influential symptoms in anxiety networks, potentially driving the development and persistence of anxiety disorders [55]. That said, our findings are the first to use network analysis to elucidate the strong potential influence of discrete states of sadness and stress on numerous discrete PA emotions in a clinical sample.

### 4.2 Model 2: discrete emotions and perceived social connection in anxiety and depression

*Sad*, *love*, and *serenity* were central bridge nodes in Model 2. Notably, *sad* and *serenity* likely emerged as strong bridge nodes due to their associations with opposing affective communities; however, they were both weakly associated with the social connection community.

#### 4.2.1 Embarrassment links NA and social connection

The NA node *embarrassed* demonstrated a strong negative cross-community edge weight with *SC1* (‘I feel comfortable in the presence of strangers’). Regarding this association, the existing literature has pointed to an increase in the experience of ‘embarrassing’ social interactions among those with social anxiety disorder and/or major depressive disorder [57]. Our findings add to this notion, highlighting a negative association between feeling embarrassed and experiencing comfort around strangers. These results, paired with previous findings that social anxiety disorder is often precipitated by a negative social event (e.g., harassment, teasing; [58]), may be indicative of a negative feedback loop wherein feeling embarrassed or self-conscious promotes social discomfort, which in turn fuels subsequent negative emotions surrounding social interactions.

#### 4.2.2 Love links PA and social connection

The emergence of *love* as a central bridge item linking PA to the social connection community suggests that this emotion may be a key mechanism through which positive emotionality fosters interpersonal closeness. In our network, *love* was directly connected to the SCS-R nodes *I am able to connect with other people*, *my friends feel like family*, *I feel understood by the people I know*, and *I feel close to people*. These results highlight the potential role of love in translating internal positive states into perceptions of social connectedness. This pattern aligns with Fredrickson’s view of love as the most ubiquitous and consequential positive emotion, arising when positive emotions are experienced in safe interpersonal contexts [33, [59]. Moreover, the association observed between *love* and *feeling understood* suggests a potential reciprocal dynamic: feeling understood in a social context may create the interpersonal safety necessary for love to arise, and experiences of love may subsequently reinforce feelings of closeness and mutual understanding [60].

The link between PA and heightened social connectedness has also been observed in clinical interventions such as Amplification of Positivity (AMP), where increases in PA uniquely predicted subsequent increases in connectedness and increases in connectedness later predicted increases in PA [10], [61]. Taken together, these findings support the notion of upward spirals of positive emotion [58], suggesting that interventions aiming to increase PA may capitalize on this feedback loop by fostering positive emotions not just independently, but ideally in the context of safe interpersonal relationships.

### 4.3 Model 3: discrete emotions and perceived social disconnection in anxiety and depression

#### 4.3.1 Guilt and shame link NA to social disconnection

In our third model, the NA node *guilt/shame* exhibited the strongest overall EI and shared a notable positive association with *SC13* (‘Even around people I know, I don’t feel that I really belong’), indicating a potential reciprocal feedback loop where negative self-evaluation reinforces a poor sense of belonging. This finding aligns with theories suggesting that shame often leads to social withdrawal and the perception of oneself as fundamentally “unfit” for social connection [62]. Taken together with our finding that *guilt/shame* had the strongest overall EI among the NA community, this suggests that guilt and shame may serve as primary drivers of both internal distress and interpersonal alienation.

#### 4.3.2 Love as a contingent state of interpersonal connection

In Model 3, *love* emerged as a key node linking PA to the social disconnection community via its negative associations with *SC11* (‘Even among my friends, there is no sense of brother/sisterhood’) and *SC14* (‘I have little sense of togetherness with my peers’). These findings coincide with Fredrickson’s theory that love is cultivated when positive emotions are experienced in the context of safe interpersonal relationships [33, [59]. If such safe, genuine interpersonal relationships do not exist, feelings of love may be unlikely to emerge. Consequently, clinical interventions prioritizing the depth and quality of one’s interpersonal relationships may be essential to create the conditions under which love can flourish. Developing such high-quality connections can foster positivity resonance–the shared experience of positive affect, mutual care, and behavioral synchrony–which is theorized to be particularly powerful in promoting health and well-being [63]. One recent study found that perceived positivity resonance was positively associated with optimal mental health and negatively associated with depressive symptoms [64]. Altogether, these results underscore the potential value of promoting high-quality social connections for individuals with anxiety and depression.

## 5 Limitations and future directions

Our study findings should be considered alongside the following limitations. First, our cross-sectional design precludes causal inference and limits our ability to determine the temporal stability of the observed network structures. Future research utilizing longitudinal designs or Ecological Momentary Assessment (EMA) would inform whether ‘influential’ nodes remain stable across time. Nevertheless, our results generated causal hypotheses to be tested in future studies. In particular, intervention research could test whether specifically targeting sadness results in increased PA (especially among directly linked positive emotions like joy, interest, amusement, and serenity) compared to targeting other negative valence emotions like fear. Similarly, future work may explore whether increasing love enhances specific feelings of connectedness like feeling understood, or whether targeting embarrassment and self-consciousness alleviates feelings of discomfort around strangers.

Second, the absence of a healthy control group limits our ability to determine whether the observed network structure is unique to individuals with clinically elevated anxiety and depression. Access to SCS-R and mDES data from healthy samples would allow for direct comparisons, offering insight into how discrete emotions and social connectedness indicators interact differently in clinical versus non-clinical populations. Examining potential differences in the way discrete emotions associate with one another across these groups could have important implications for how we understand mental disorders like anxiety and depression. Additionally, future research should examine whether the observed network structures and central nodes differ across diagnostic groups or whether they are transdiagnostic.

Lastly, the generalizability of our findings may be constrained by the characteristics of our sample, which were determined by the eligibility criteria of the parent clinical trials (e.g., the upper age limit was set to minimize confounding of functional MRI outcomes due to age-related neurocognitive changes). Consequently, our results may not fully represent the emotional and social network structures of different populations (e.g., older adults, youth).

## 6 Conclusion

The present study provides a fine-grained examination of the relationships between discrete positive emotions, negative emotions, indicators of social connection, and indicators of social disconnection among people experiencing clinically elevated anxiety or depression. By applying network analysis at the individual item level, this work clarifies how certain emotions interrelate and contribute to the broader affective profiles common in those with such diagnoses. Importantly, the findings suggest that cultivating hope and alleviating sadness may serve as valuable treatment targets for enhancing overall positive affect. Moreover, mitigating negative self-perceptions like guilt and shame may have positive implications for improving one’s sense of belonging within social contexts. Lastly, the results indicate that improving the depth and quality of one’s existing relationships while fostering positive emotions within such interpersonal contexts may promote feelings of love. This study represents an initial step toward a more comprehensive understanding of the nuanced interplay between positive affect, negative affect, and social connectedness.

## Footnotes

Footnote 1: The OASIS and PHQ-9 were not administered until partway through data collection for study 1 (NCT02136212).

## Funding

This research was supported by grants awarded to Charles T. Taylor from the 10.13039/100000025National Institute of Mental Health (R00MH090243, R61MH113769 and R33MH113769) and the 10.13039/100000874Brain and Behavior Research Foundation (21695). The project described was partially supported by the 10.13039/100000002National Institutes of Health, Grant UL1TR000100 of CTSA funding prior to August 13, 2015 and Grant ULTR001442 of 10.13039/100016220CTSA funding beginning August 13, 2015 and beyond, as well as the 10.13039/100000026National Institute on Drug Abuse under Award no. 1K23DA062034 awarded to Amanda C. Collins.

## Declaration of Competing Interest

Charles T. Taylor declares that in the past 3 years he has been a paid consultant for Bionomics, atai Life Sciences, and Engrail Therapeutics, and receives payment for editorial work for UpToDate. Ryan C. Shriver, Madeleine M. Rassaby, and Amanda C. Collins declare no conflicts of. All procedures performed involving human participants were in accordance with the ethical standards of the University of California San Diego Human Research Protection Program and with the Code of Ethics of the World Medical Association (Declaration of Helsinki).
