# Supplementary material for: Network analyses of discrete emotions and social connectedness indicators in anxiety and depressive disorders
Source: J Mood Anxiety Disord. 2026 Apr 1;14:100177. doi: 10.1016/j.xjmad.2026.100177 (PMC13101674; doi:10.1016/j.xjmad.2026.100177)
Supplement: Supplementary file 1 — Supplementary material [file mmc1.docx]

**Supplementary Materials**

*Network analyses of discrete emotions and social connectedness indicators in anxiety and depressive disorders*

**Combined Samples - Social Functioning Comparisons**

To assess potential bias from varying eligibility criteria across the five parent trials, we ran multiple analyses comparing the earlier trials (1-3) to the later trials (4-5), the latter of which specifically recruited participants with disrupted social functioning and social disconnection. An independent samples t-test on SCS-R scores revealed no significant difference between cohorts, t(357) = 0.26, *p* = .759 (Samples 1-3: M = 60.58, SD = 14.22; Samples 4-5: M = 60.19, SD = 13.84, Cohen’s d = .03). Furthermore, SDS-S outcomes indicated that both cohorts fell within the moderate-to-marked degree of social impairment, with the earlier trials reporting statistically higher social impairment t(347) = 3.12, *p* = .002 (Samples 1-3: M = 6.88, SD = 1.89) than the targeted trials (Samples 4-5: M = 6.23, SD = 1.98, Cohen’s d = .34).

**Table S1**

*20 mDES emotion descriptors*

| Emotion (node name) | Descriptor |
| --- | --- |
| Amused | How often have you felt amused, fun-loving, or silly? |
| Awe | How often have you felt awe, wonder, or amazement |
| Grateful | How often have you felt grateful, appreciative, or thankful |
| Hope | How often have you felt hopeful, optimistic, or encouraged |
| Inspired | How often have you felt inspired, uplifted, or elevated |
| Interested | How often have you felt interested, alert, or curious |
| Joy | How often have you felt joyful, glad, or happy |
| Love | How often have you felt love, closeness, or trust |
| Proud | How often have you felt proud, confident, or self-assured |
| Serenity | How often have you felt serene, content, or peaceful |
| Angry | How often have you felt angry, irritated, or annoyed |
| Ashamed | How often have you felt ashamed, humiliated, or disgraced |
| Contempt | How often have you felt contemptuous, scornful, or disdainful |
| Disgust | How often have you felt disgust, distaste, or revulsion |
| Embarrassed | How often have you felt embarrassed, self-conscious, or blushing |
| Guilt | How often have you felt guilty, repentant, or blameworthy |
| Hatred | How often have you felt hate, distrust, or suspicion |
| Sad | How often have you felt sad, downhearted, or unhappy |
| Scared | How often have you felt scared, fearful, or afraid |
| Stressed | How often have you felt stressed, nervous, or overwhelmed |

**Figure S1**

*Model 1 Edge Accuracy Test
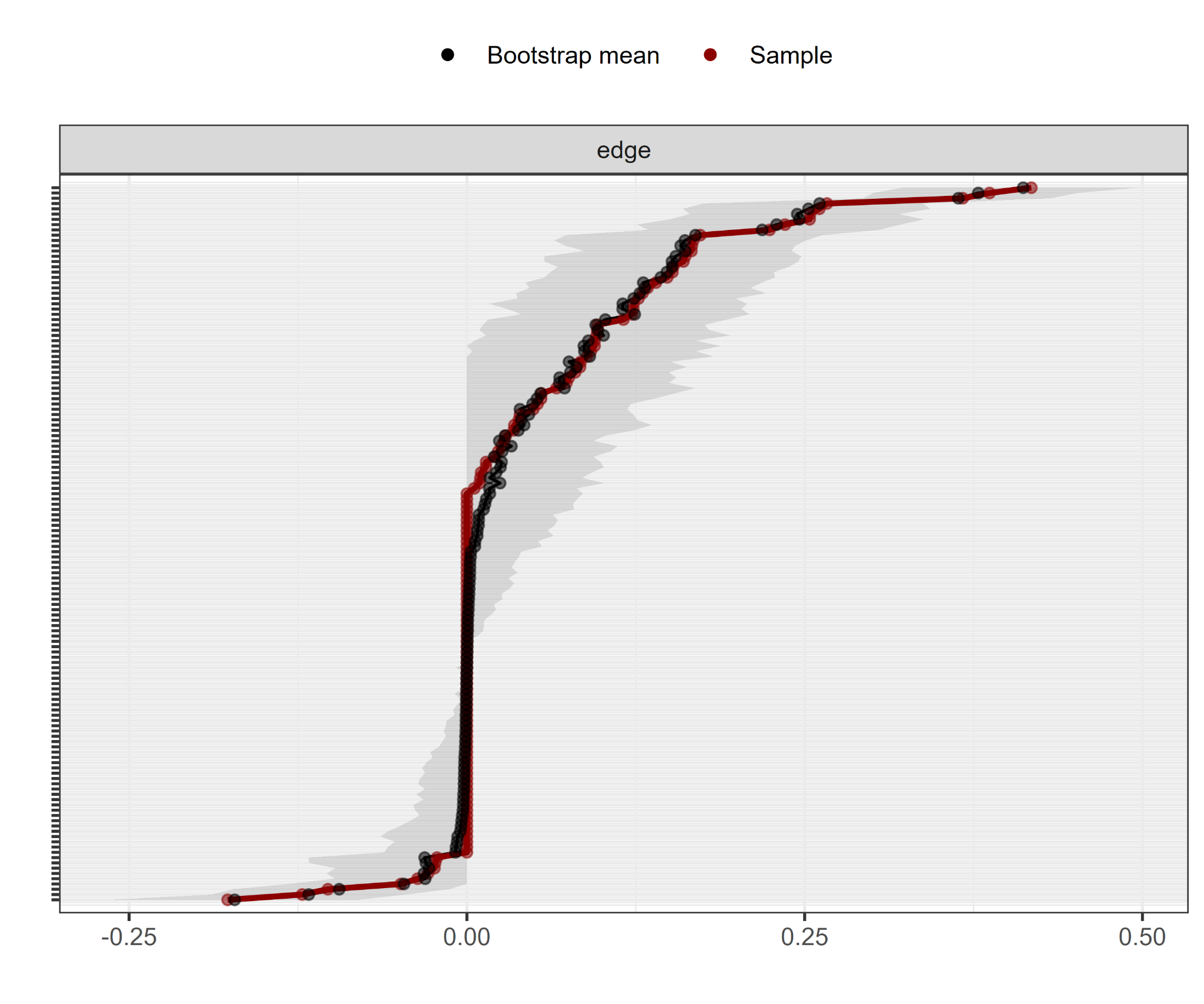
*

**Figure S2**

*Model 1 EI Difference Test*

*
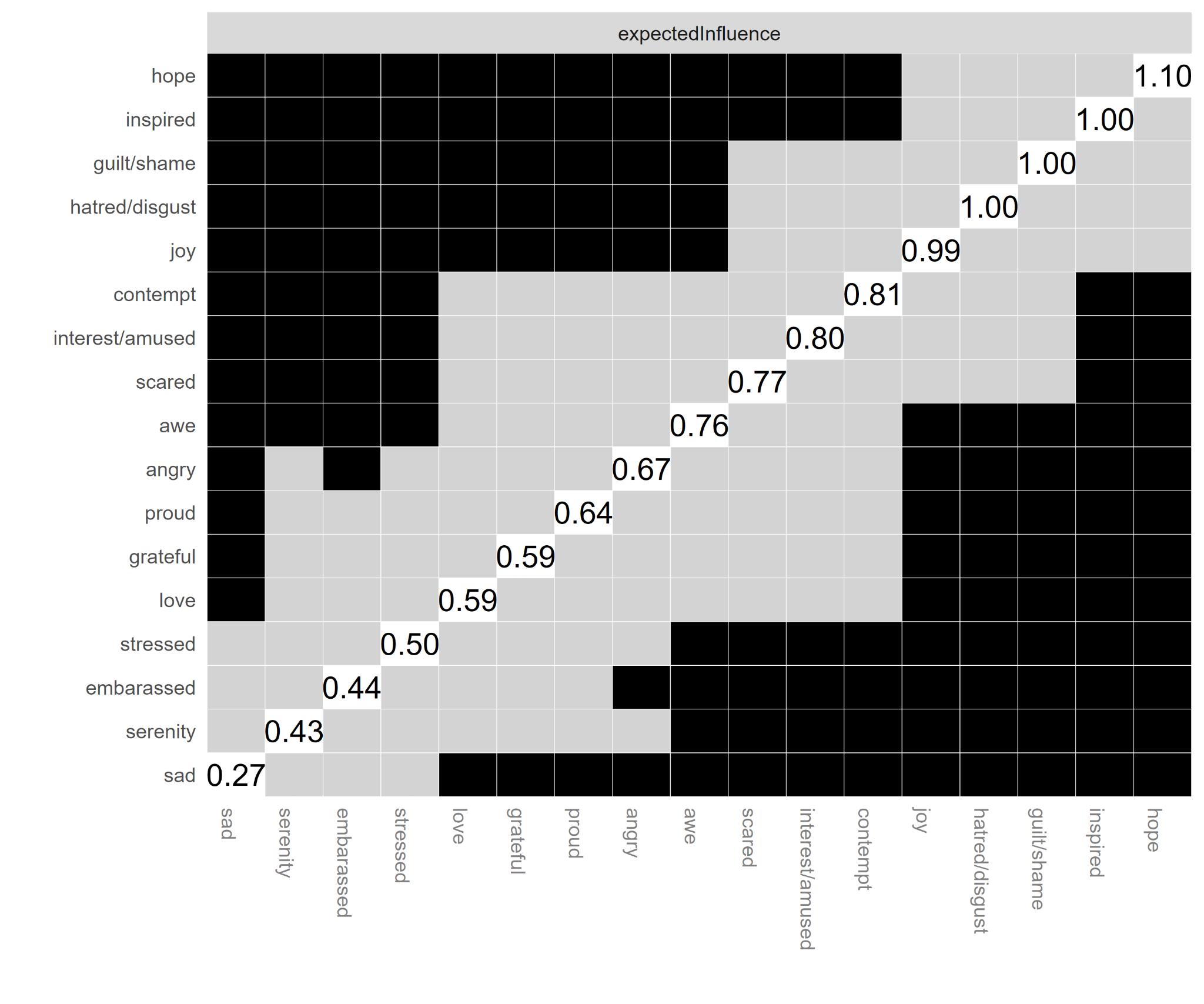
*

**Figure S3**

*Model 1 bEI Difference Test*

**
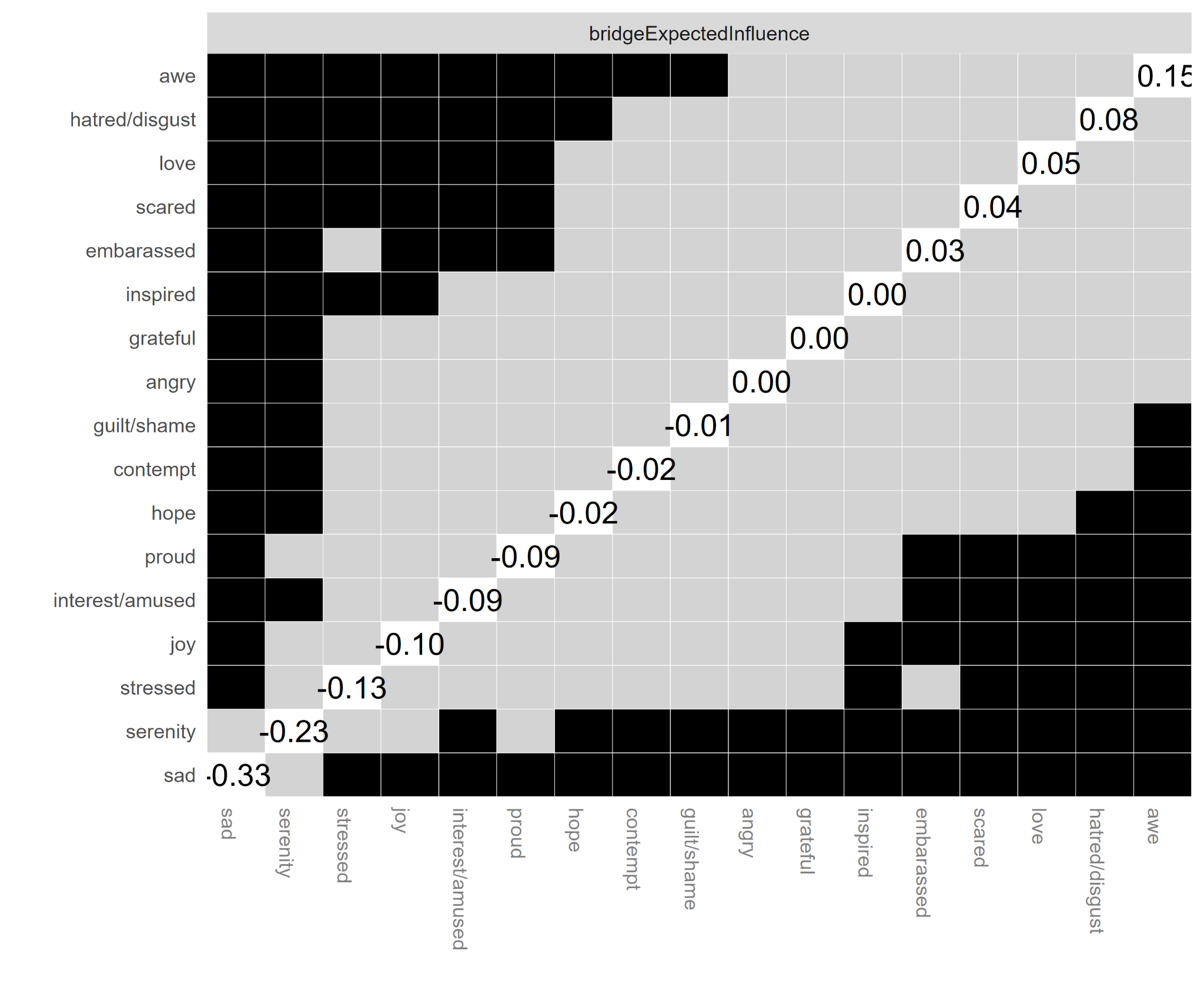
**

**Figure S4**

*Model 1 Edge Stability*

**
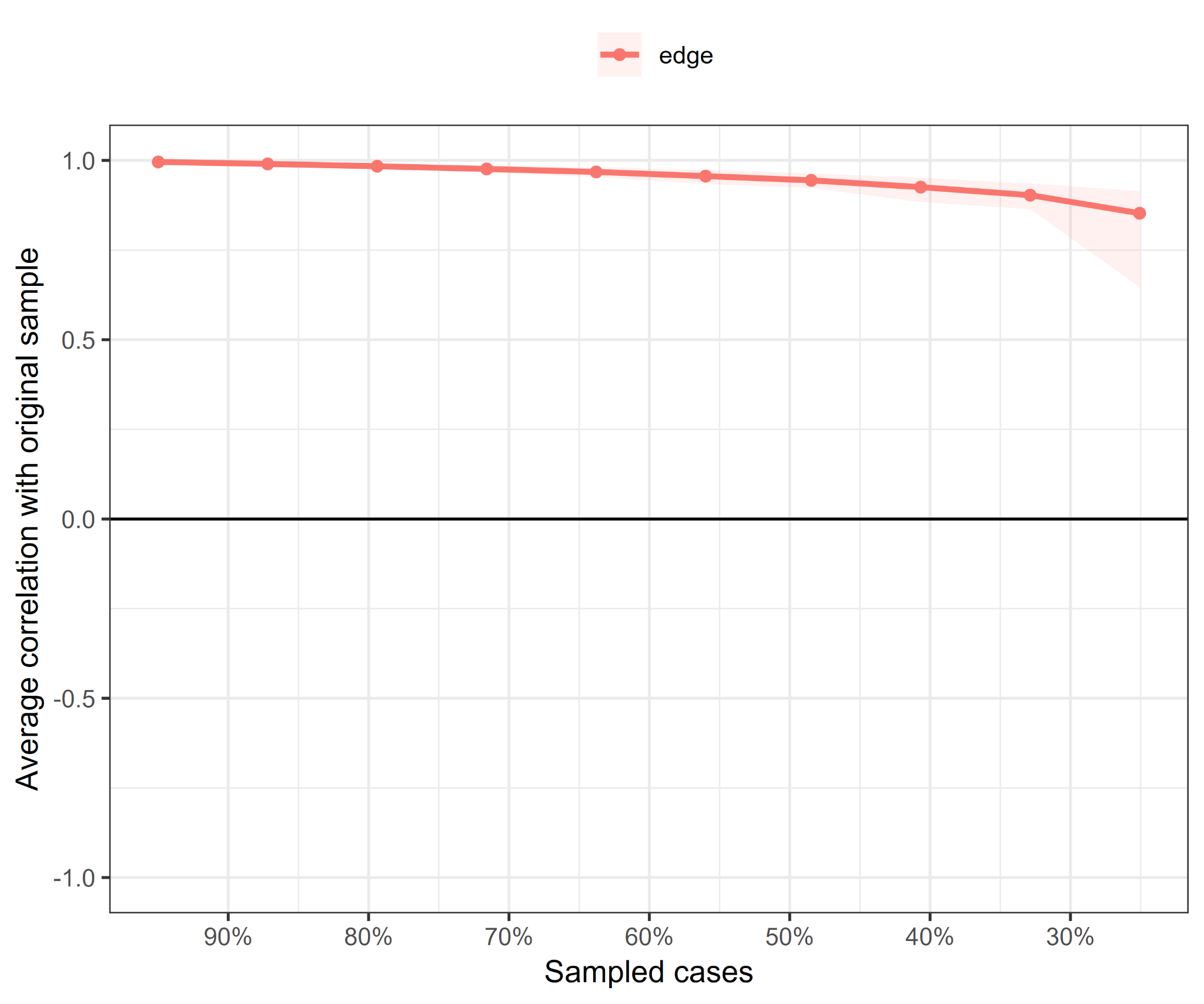
**

**Figure S5**

*Model 1 EI Stability*


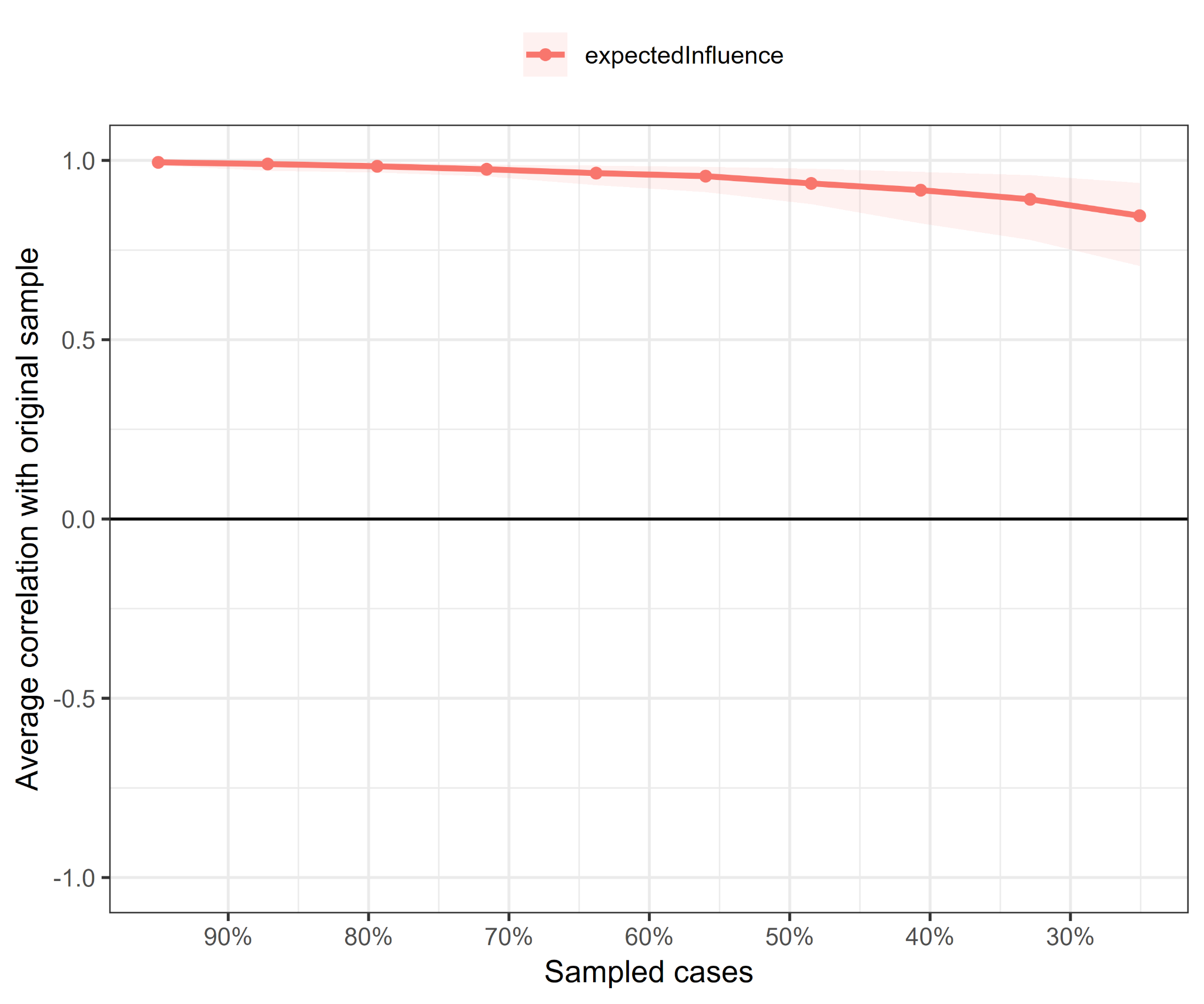


**Figure S6**

*Model 1 bEI Stability*

**
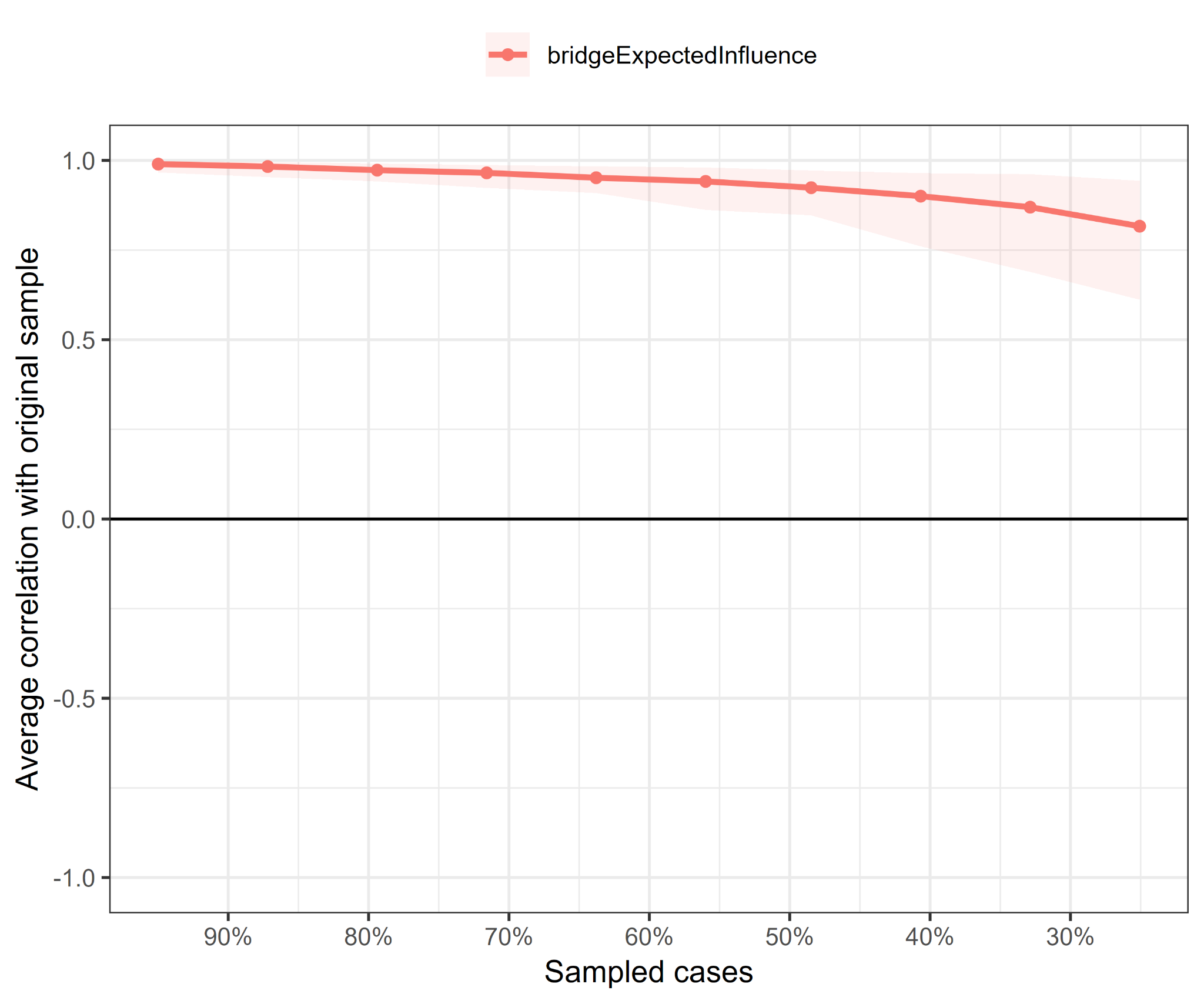
**

**Figure S7**

*Model 2 Edge Accuracy Test*


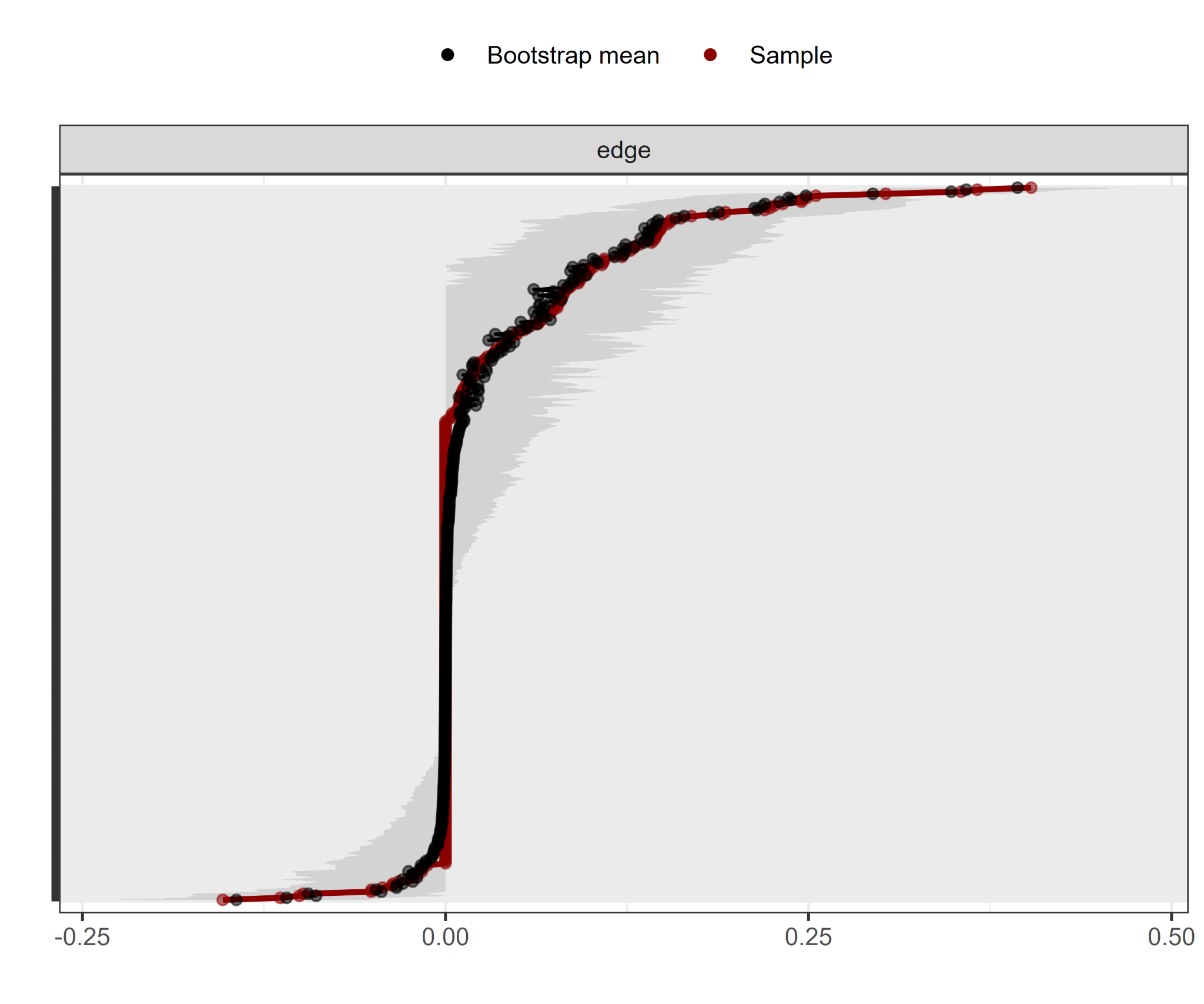


**Figure S8**

*Model 2 EI Difference Test
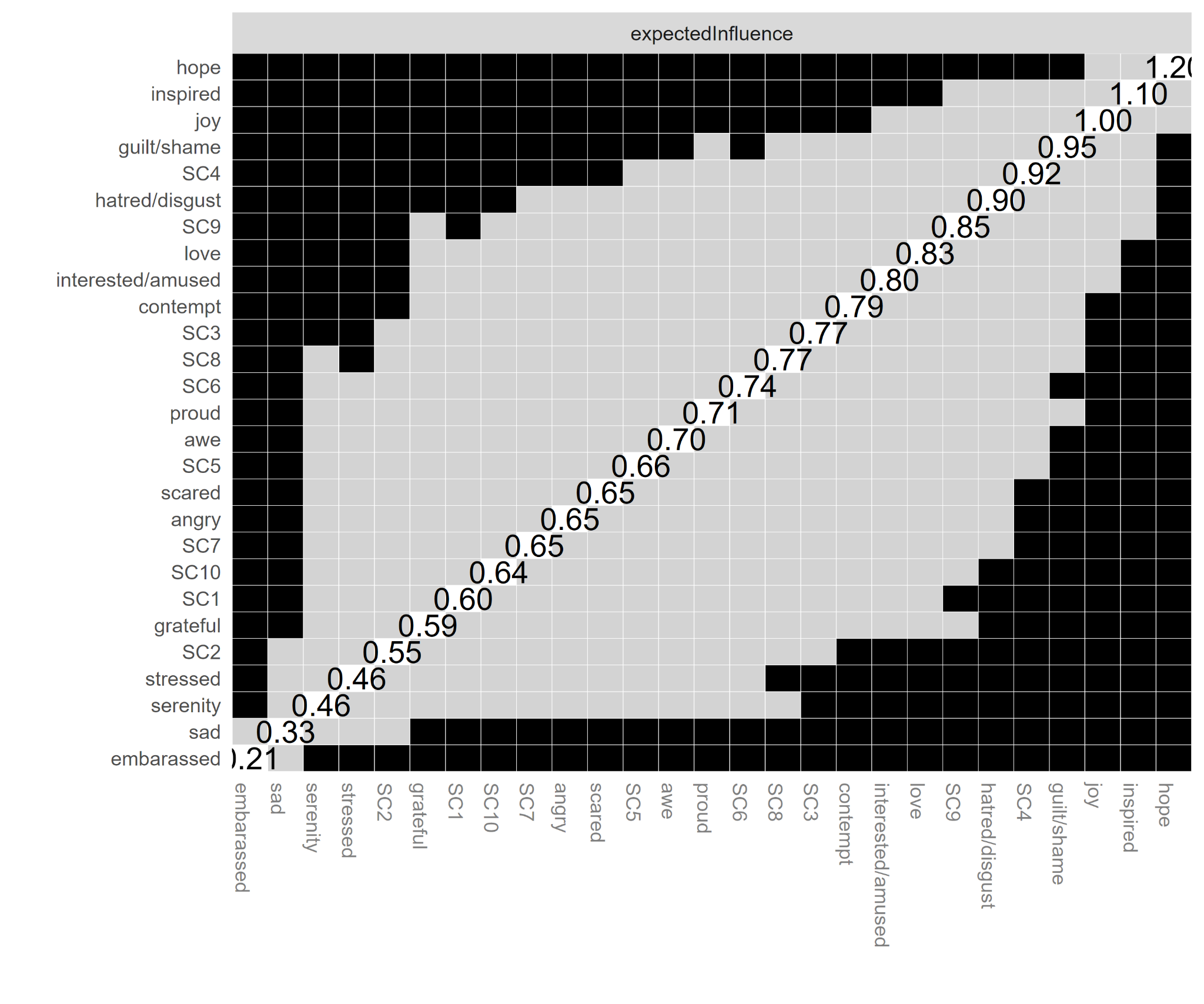
*

**Figure S9**

*Model 2 bEI Difference Test*


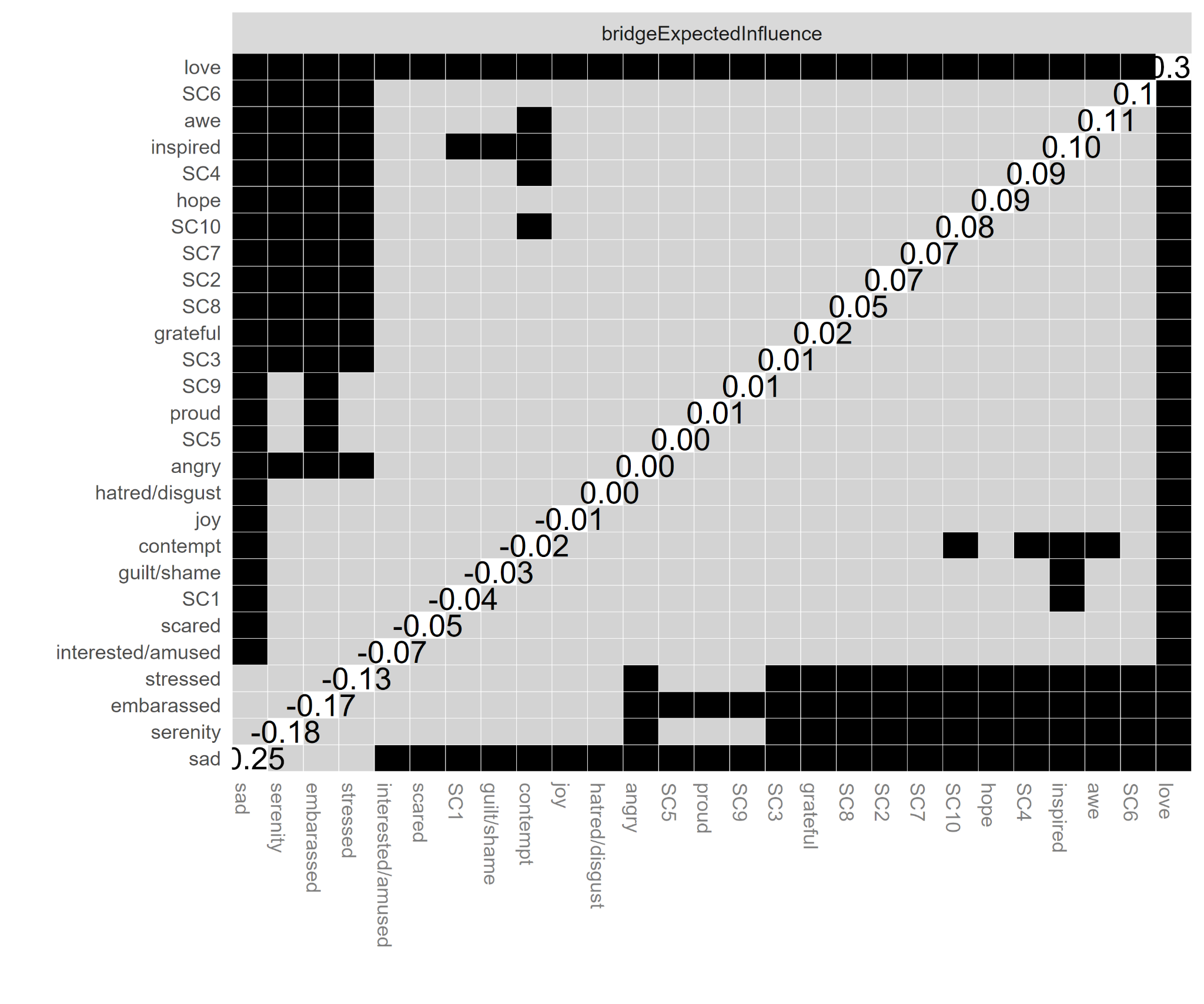


**Figure S10**

*Model 2 Edge Stability*

**
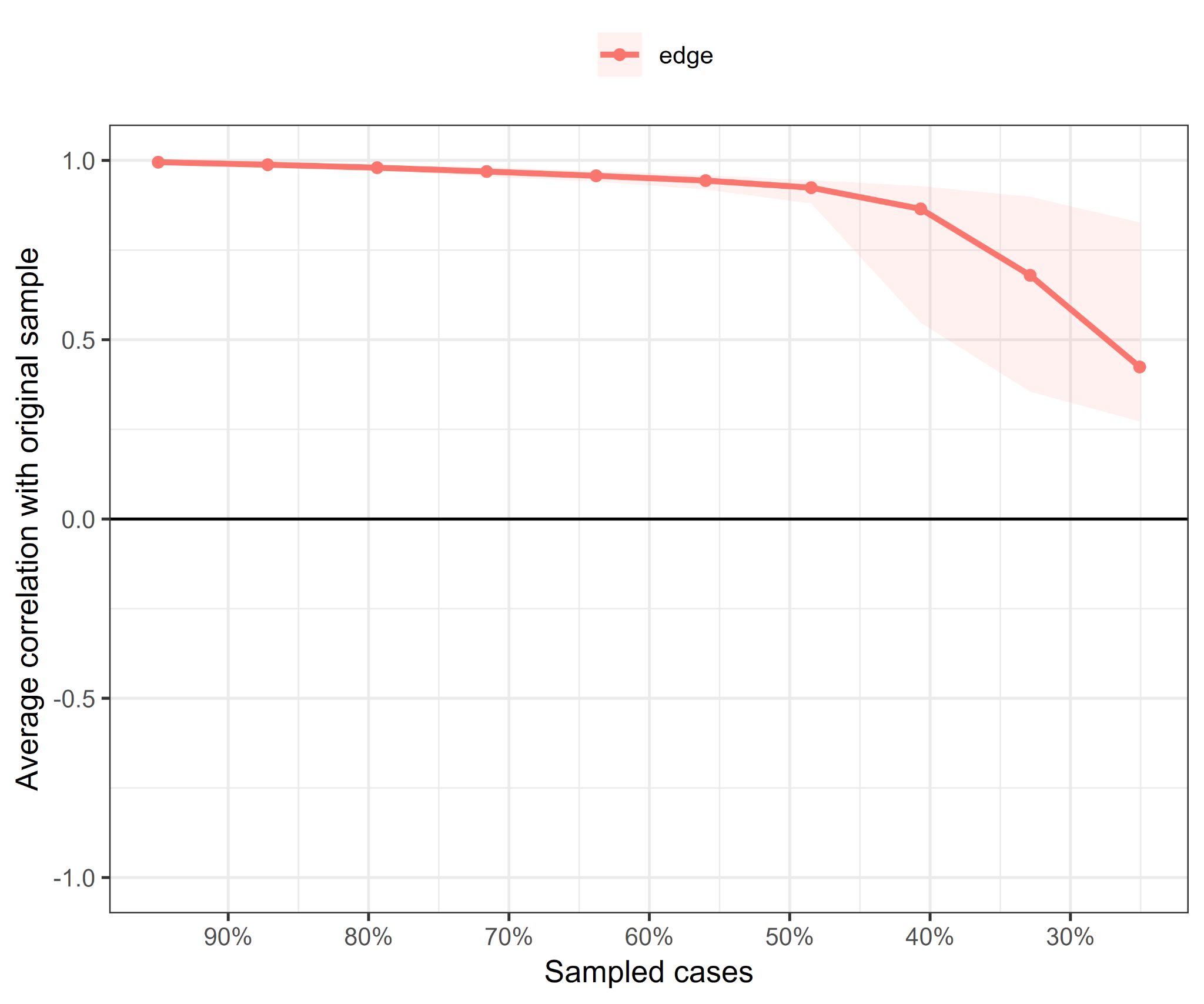
**

**Figure S11**

*Model 2 EI Stability*

**
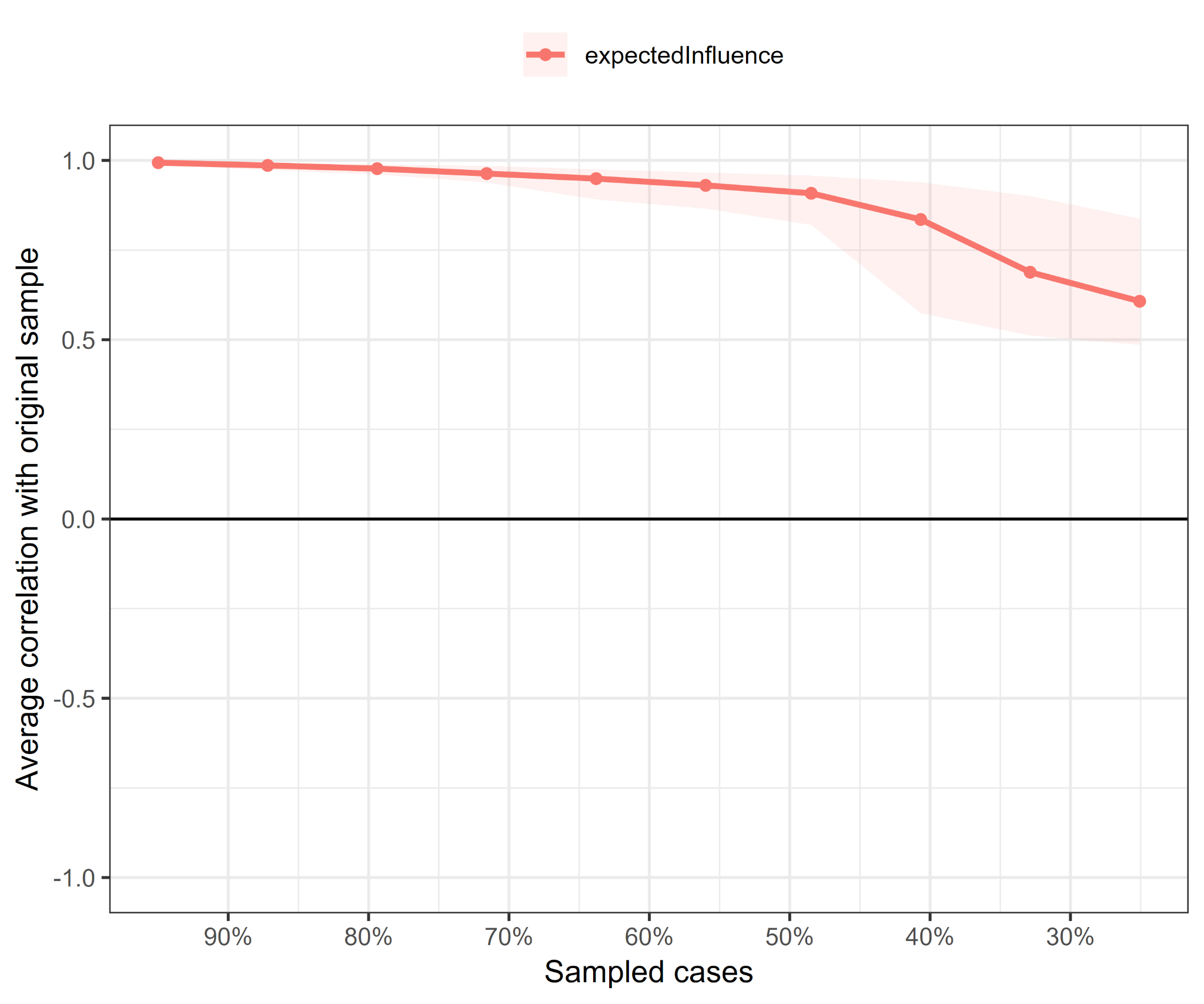
**

**Figure S12**

*Model 2 bEI Stability***
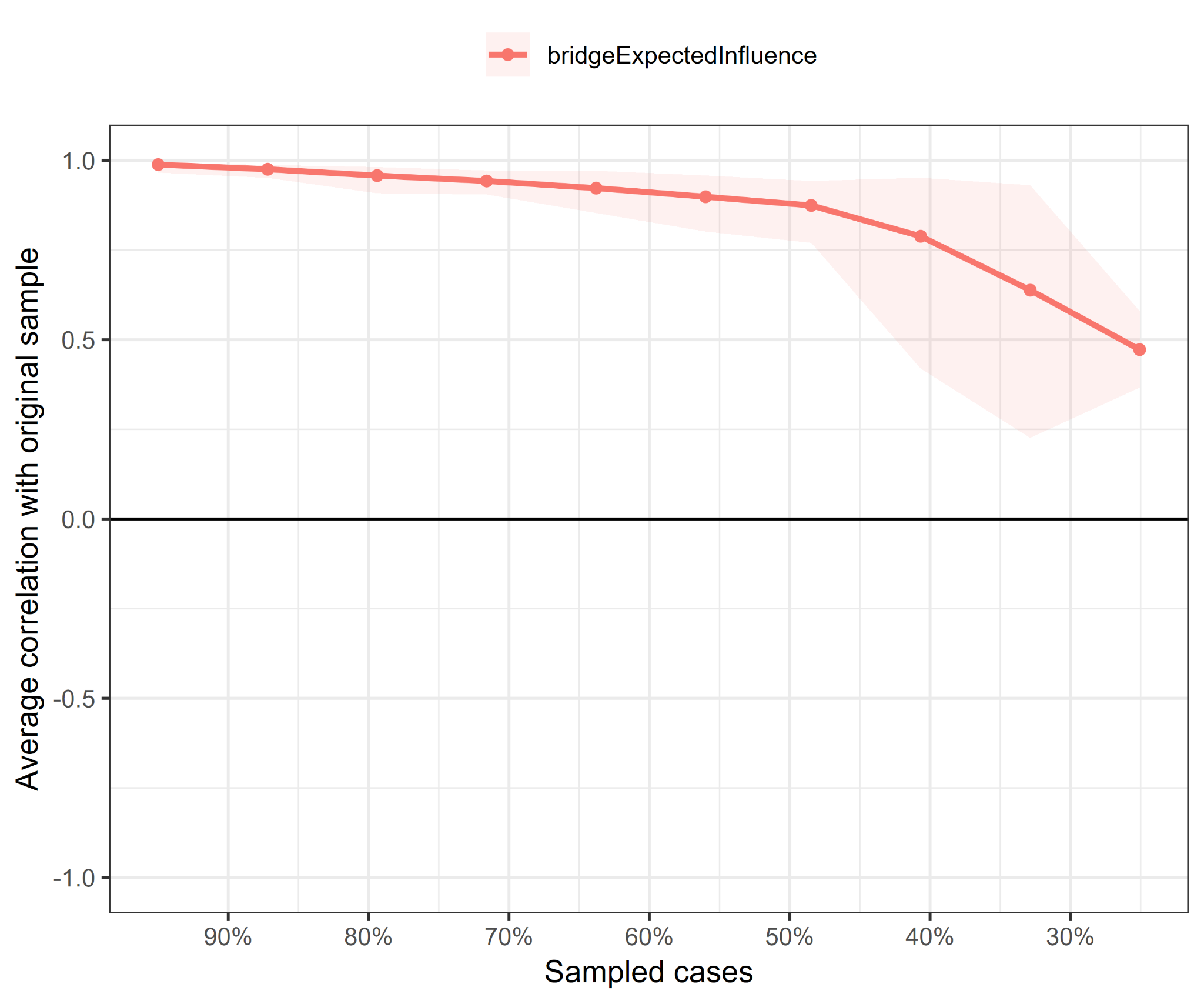
**

**Figure S13**

*Model 3 Edge Accuracy Test*


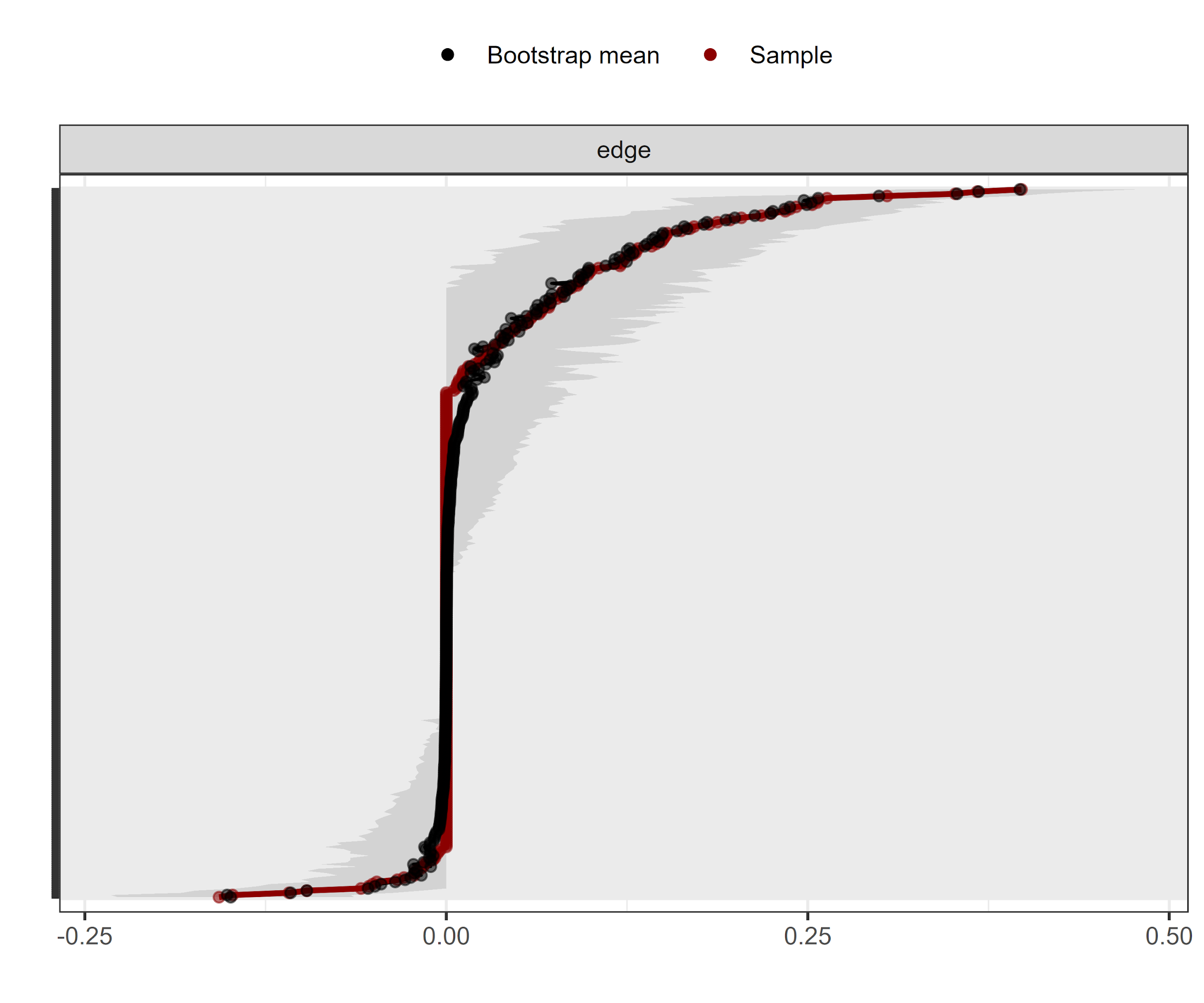


**Figure S14**

*Model 3 EI Difference Test
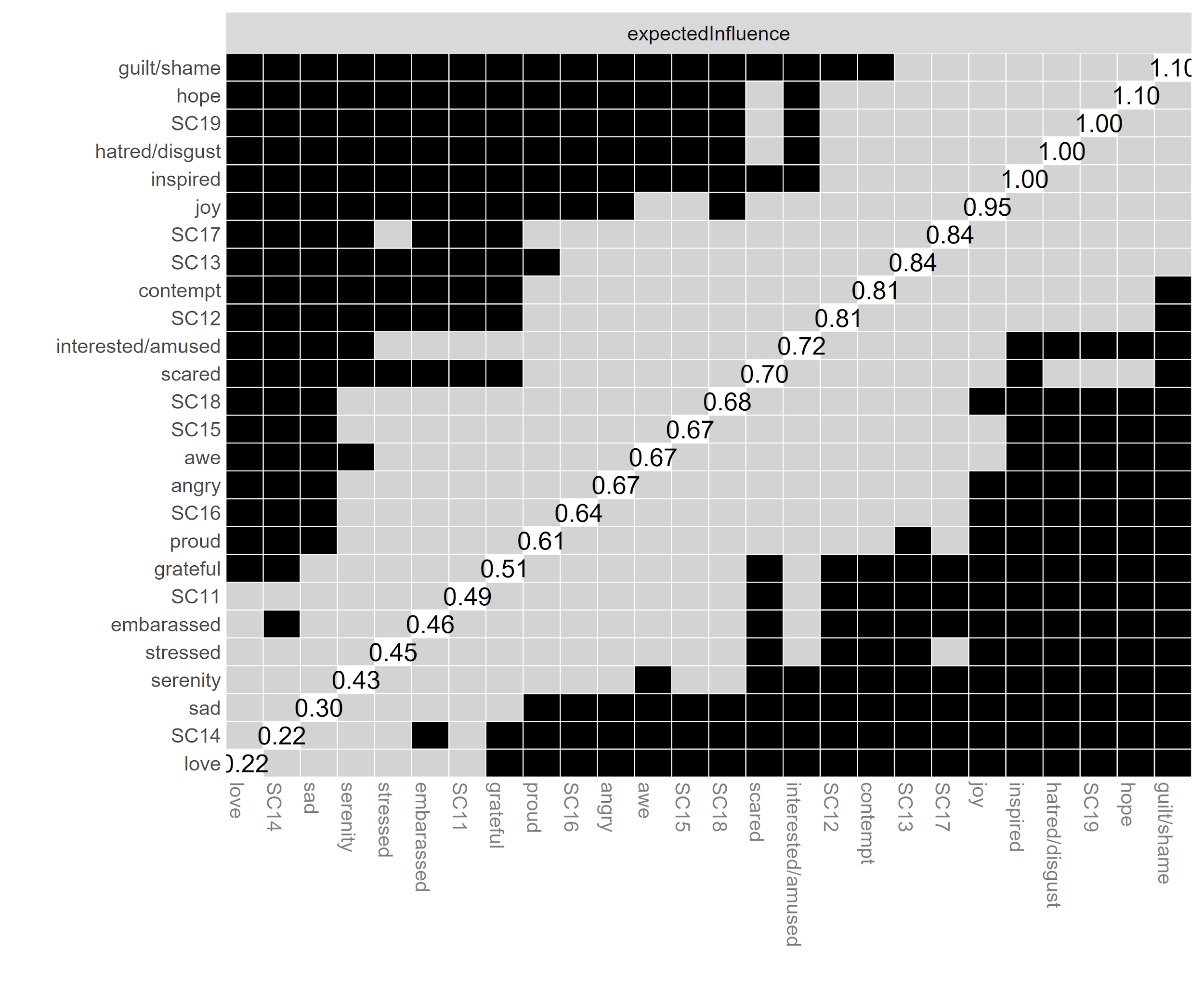
*

**Figure S15**

*Model 3 bEI Difference Test*


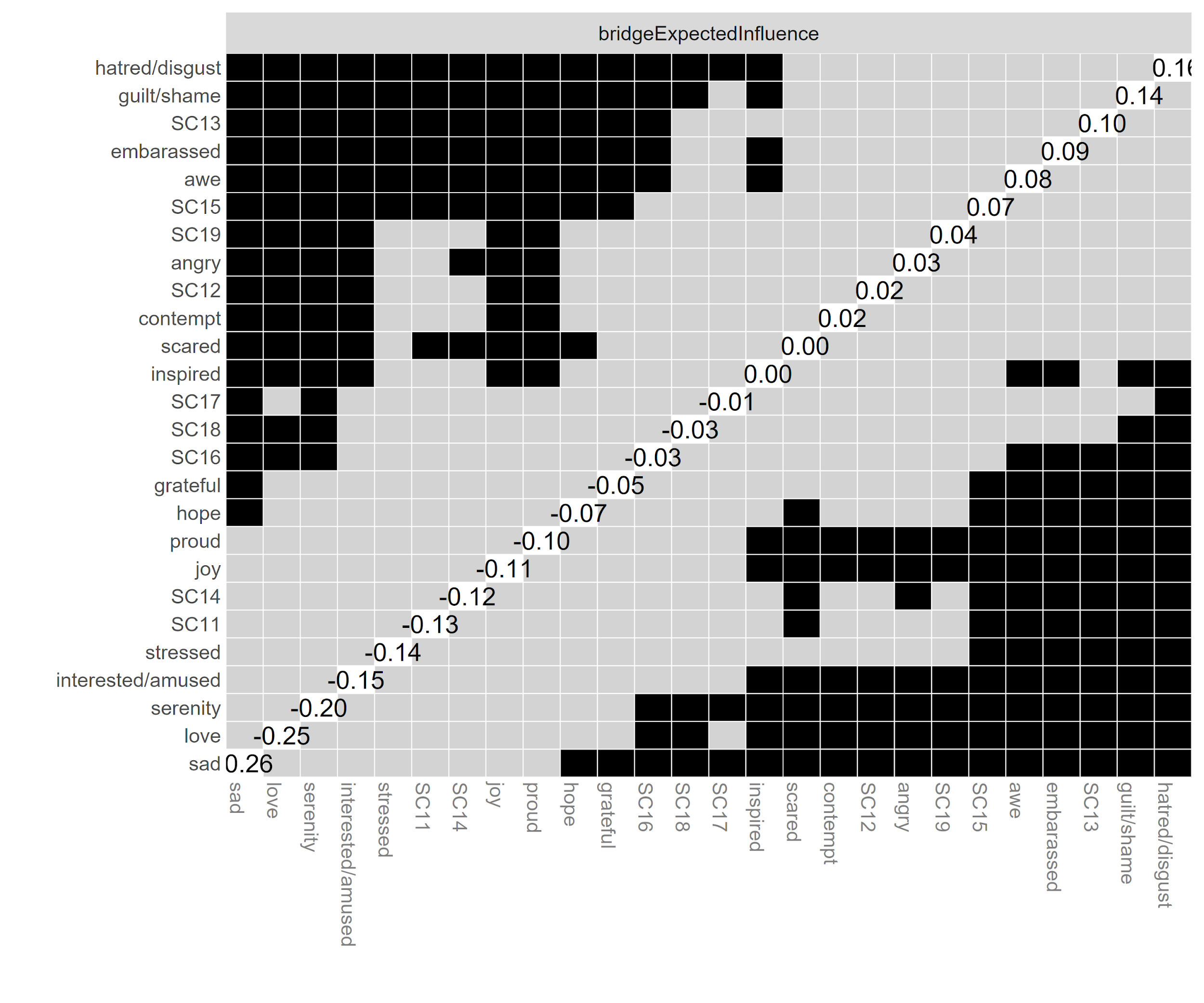


**Figure S16**

*Model 3 Edge Stability*

**
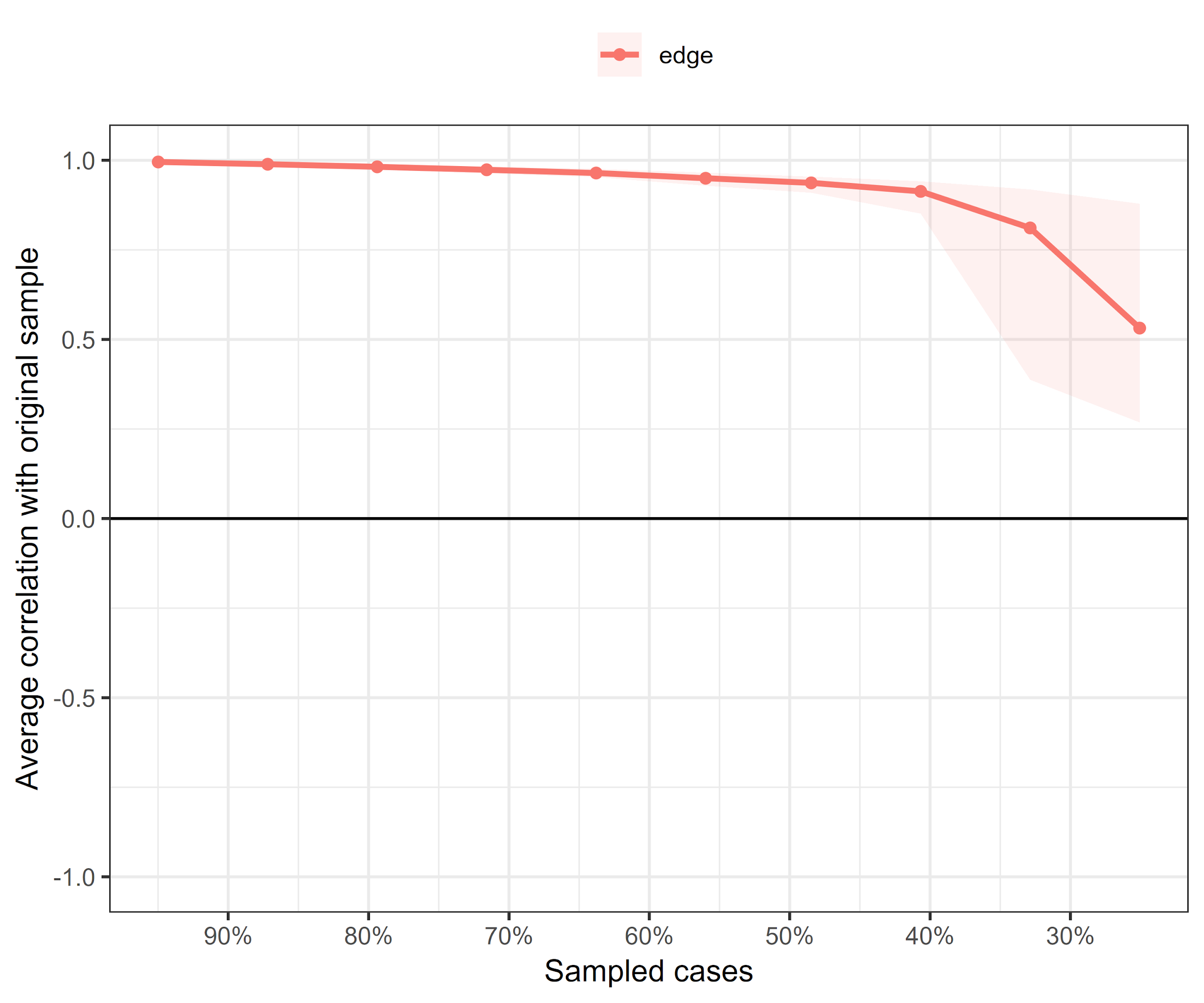
**

**Figure S17**

*Model 3 EI Stability*

**
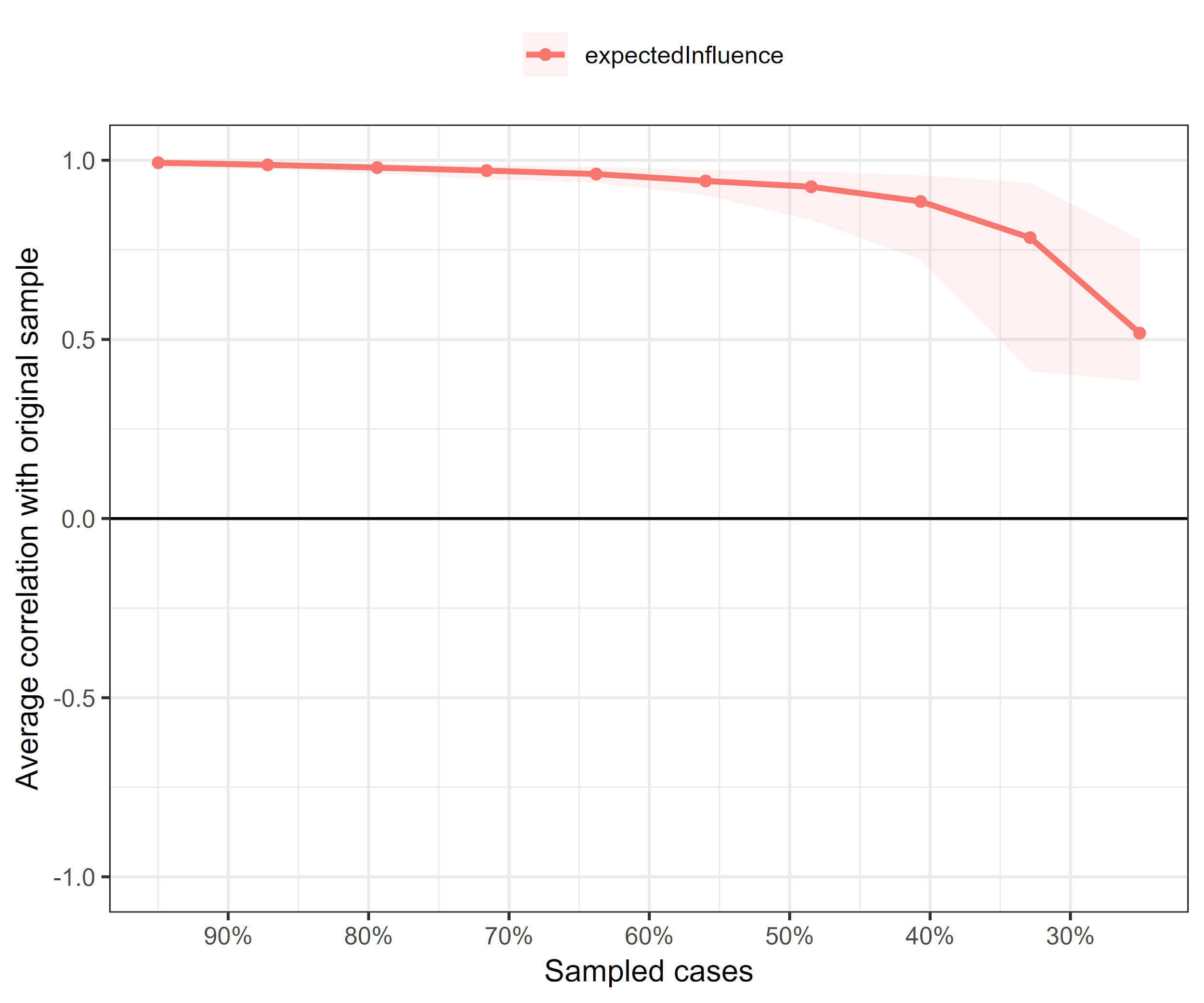
**

**Figure S18**

*Model 3 bEI Stability*

**
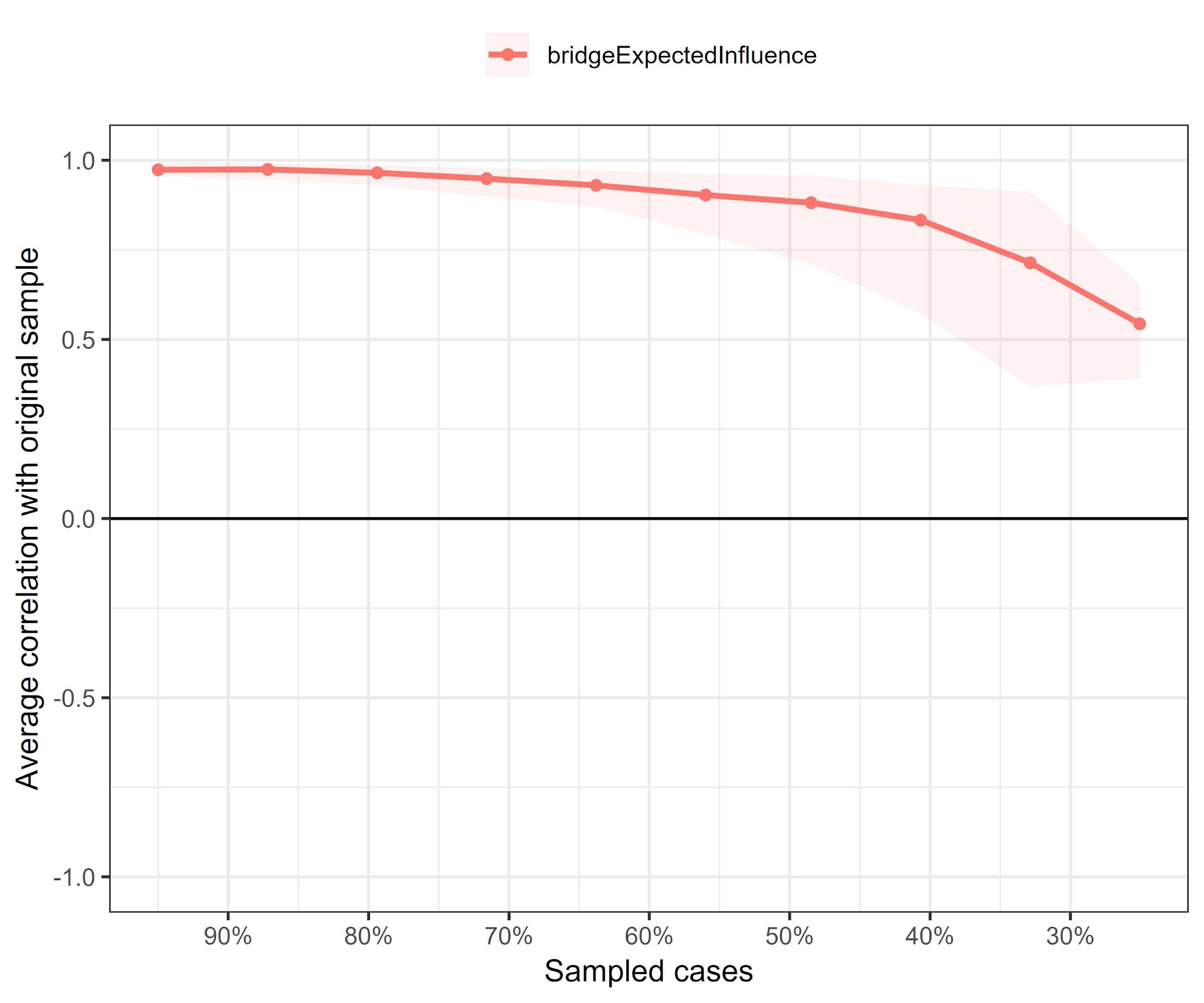
**
